# Maturation of UTR-Derived sRNAs Is Modulated during Adaptation to Different Growth Conditions

**DOI:** 10.3390/ijms222212260

**Published:** 2021-11-12

**Authors:** Daniel-Timon Spanka, Gabriele Klug

**Affiliations:** Institute of Microbiology and Molecular Biology, Justus Liebig University Giessen, Heinrich-Buff-Ring 26-32, 35392 Giessen, Germany; daniel-timon.spanka@mikro.bio.uni-giessen.de

**Keywords:** UTR-derived sRNA, sRNA processing and maturation, ribonucleases, RNase E, *Rhodobacter sphaeroides*, Alphaproteobacteria

## Abstract

Small regulatory RNAs play a major role in bacterial gene regulation by binding their target mRNAs, which mostly influences the stability or translation of the target. Expression levels of sRNAs are often regulated by their own promoters, but recent reports have highlighted the presence and importance of sRNAs that are derived from mRNA 3′ untranslated regions (UTRs). In this study, we investigated the maturation of 5′ and 3′ UTR-derived sRNAs on a global scale in the facultative phototrophic alphaproteobacterium *Rhodobacter sphaeroides*. Including some already known UTR-derived sRNAs like UpsM or CcsR1-4, 14 sRNAs are predicted to be located in 5 UTRs and 16 in 3′ UTRs. The involvement of different ribonucleases during maturation was predicted by a differential RNA 5′/3′ end analysis based on RNA next generation sequencing (NGS) data from the respective deletion strains. The results were validated in vivo and underline the importance of polynucleotide phosphorylase (PNPase) and ribonuclease E (RNase E) during processing and maturation. The abundances of some UTR-derived sRNAs changed when cultures were exposed to external stress conditions, such as oxidative stress and also during different growth phases. Promoter fusions revealed that this effect cannot be solely attributed to an altered transcription rate. Moreover, the RNase E dependent cleavage of several UTR-derived sRNAs varied significantly during the early stationary phase and under iron depletion conditions. We conclude that an alteration of ribonucleolytic processing influences the levels of UTR-derived sRNAs, and may thus indirectly affect their mRNA targets.

## 1. Introduction

Most bacteria live in environments that are subjected to changes in available nutrients, oxygen concentration, light conditions, temperature, and other parameters. In order to survive these changes and defend harmful stress conditions, bacteria have to adjust several layers of gene regulation and thus their physiology. This mostly goes along with massive changes of the transcriptome (e.g.,: [1,2,3]), but it is now well recognized that regulation at the post-transcriptional level also plays a crucial role in bacterial adaptation (e.g.,: [4,5]). Small RNAs affect regulation mostly at the post-transcriptional level, and make important contributions to the adaptation to stress conditions in bacteria ([6,7]; as reviewed in [8]). The function of several sRNAs in stress adaptation was investigated in the facultative phototroph *Rhodobacter sphaeroides*. This bacterium can perform aerobic or anaerobic respiration, fermentation or anoxygenic photosynthesis. Since the formation of photosynthetic complexes in the presence of high oxygen levels generates singlet oxygen, the formation of photosynthetic complexes and of defense systems is regulated by oxygen concentration, light intensity and the concentration of reactive oxygen species (e.g., [9,10,11,12,13]; reviewed in [14]). Some of the sRNAs involved in this regulation are derived from the 5′ or 3′ UTRs of precursor transcripts by processing. SorX is derived from the 3′ UTR of the *ompR-1* mRNA (*RSP_0847*) by RNase E-mediated cleavage [15,16]. Its level strongly increases in response to several stresses, and by targeting the mRNA for a subunit of a spermidine transporter it counteracts oxidative stress [15]. The oxidative stress-induced CcsR1-4 RNAs are generated by processing of the 3′ UTR of the *ccaF1* mRNA (*RSP_6037*) and modulate the C1 metabolism and the pyruvate dehydrogenase complex [17]. RNase E as well as the small CcaF1 protein have important roles in maturation of the CcsR RNAs [18]. PcrX is derived by RNase E mediated cleavage from the 3′ UTR of the polycistronic *pufBALMX* mRNA. The *pufBALMX* mRNA encodes proteins of the photosynthetic complexes and PcrX targets *pufX* and modulates the expression of *puf* genes [19]. UpsM is derived from the 5′ UTR of the *dcw* (division and cell wall) gene cluster by RNase E mediated cleavage [20] and strongly influences growth of *R. sphaeroides* by influencing the *dcw* mRNA levels in *cis* and in *trans* [21]. The expression of all these sRNAs depends on the activity of a promoter, which in case of the 3′ UTR-derived RNAs is located in front of the upstream gene. Since in addition the levels of these sRNAs depend on maturation steps, we wondered whether these maturation steps may also be influenced by environmental factors and thus contribute to regulation. To address this, we first identified further 5′ or 3′ UTR-derived sRNAs and elucidated their generation by processing. Analysis of the sRNA levels in various mutants allowed us to decipher the involvement of the RNA-binding protein Hfq and of various RNases.

Several different RNases can be found in *R. sphaeroides*, among them the highly conserved and essential endoribonuclease E (RNase E). It has a major influence on large portions of the transcriptome, since more than 15,000 cleavage sites could be identified in *R. sphaeroides* [16]. RNase E cleaves mainly at AU-rich regions in *E. coli* [22], but also in *R. sphaeroides* [16] and binds to monophosphorylated 5′ ends [23]. Furthermore, RNase E is involved in processing of sRNAs from 3′ UTRs [19,24,25,26]. RNase III is another important endonuclease that plays a role during rRNA processing [27] and mRNA turnover in *E. coli* [28,29] and *Rhodobacter* [30,31,32]. Another important enzyme in the RNA life cycle is PNPase, which acts as a 3′-to-5′ exonuclease and degrades mRNAs [33] and sRNAs [34,35]. Recent studies showed that PNPase often attacks RNA 3′ ends that were generated by endonucleases such as RNase Y in the Gram-positive *Streptococcus pyogenes* [36] or by RNase E and RNase III in the Gram-negative organism *R. sphaeroides* [37]. Besides RNase E and RNase III, RNase J1 also takes part in rRNA processing by cleaving intervening sequences of the 23S rRNA in *R. sphaeroides* [38]. Only a very few other transcripts could be identified that are processed by RNase J [39]. Furthermore, we analyzed the involvement of RppH (an RNA pyrophosphohydrolase in *E. coli*; [40]) and YbeY (involved in maturation of 16S rRNA in *E. coli*; [41]) in regard to their role in UTR-derived sRNA maturation in *R. sphaeroides*. Alongside the mentioned RNases, the RNA chaperone Hfq is necessary in many cases to ensure a functional post-transcriptional gene regulation. Hfq can assist the hybrid formation between sRNAs and their target RNAs, thus influencing the RNA stability or translation [42,43]. This mechanism is also highly relevant under stress conditions [44,45].

Our results confirmed that the levels of UTR-derived sRNAs are not only determined by the levels of transcription, but that maturation is also influenced by environmental conditions and therefore needs to be considered as another important step of regulation.

## 2. Results

### 2.1. Identification of Five Novel UTR-Derived sRNAs

**Figure 1:**
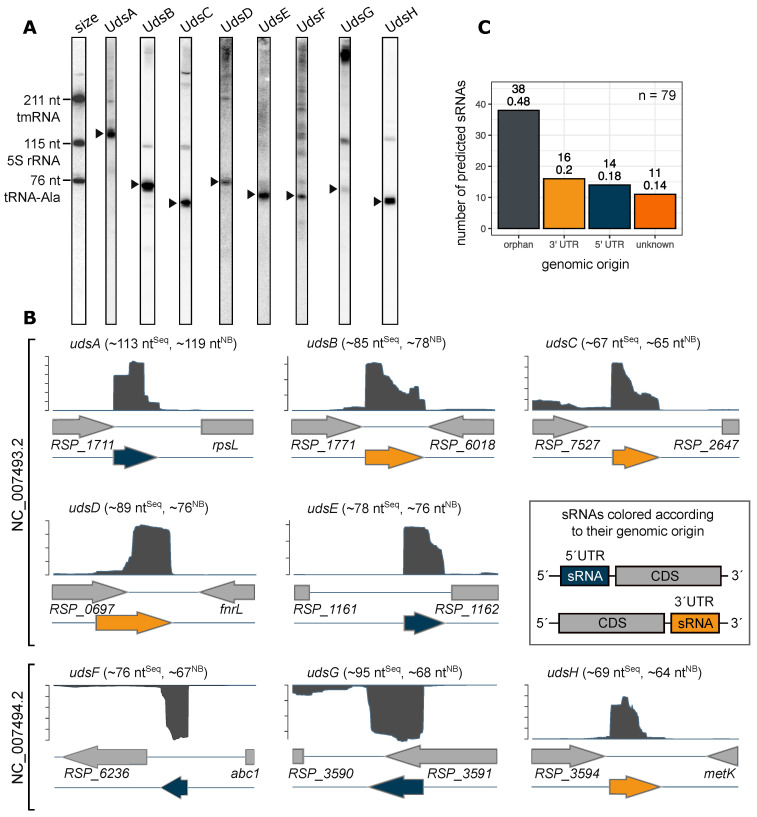
Identification of novel UTR-derived sRNAs in *Rhodobacter sphaeroides*. (**A**) 10 μg of total RNA from exponentially growing *R. sphaeroides* wild type cultures were separated on a denaturating 10% PAA gel and subsequentially blotted. Probes were directed against predicted sRNA sequences. Black triangle marks the mature sRNA. The tmRNA (211 nt), 5S rRNA (115 nt) and tRNA-Ala (76 nt) were used as an internal size standard. 5S rRNA served as loading control. Three sRNAs have previously been mentioned: UdsA (formerly IGR_RSP_1711_rpsL; [37]), UdsC (formerly the sRNA downstream of *RSP_7527*; [16]), and UdsE (formerly RSs2778; [46]). (**B**) Total read coverage of the Uds’ loci. Axis’ not to scale. Lengths were predicted by RNA sequencing (^Seq^) and northern blot analysis (^NB^). (**C**) The genomic origin of all known and predicted sRNAs in *R. sphaeroides* was prediced. Nearly 50% of all sRNAs originate from orphan genes. total *n* = 79.

Several sRNAs were shown to play a role in stress responses in *Rhodobacter sphaeroides*, including few UTR-derived sRNAs [15,17,19,20]. In this study we depict a general picture of UTR-derived sRNAs in *Rhodobacter sphaeroides*. Based on RNA-Seq datasets, we especially searched for sRNAs that are located in the 5′- or 3′-UTR of mRNA transcripts. We were able to predict the presence of five novel UTR-derived sRNAs, which could all be validated via northern blot analysis. The lengths were predicted using RNA-Seq data and confirmed on northern blots using known RNAs as size markers (Figure 1A). In addition to the five novel sRNAs, we also included three sRNAs in our analysis, which have been described previously: IGR_RSP_1711_rpsL ([37]; here renamed UdsA), the sRNA downstream of *RSP_7527* ([16]; here named UdsC), and RSs2778 ([46]; here renamed to UdsE). These eight sRNAs were named UdsA to UdsH in regard to their maturation (UTR-derived sRNA). The described Uds’ can be found on both chromosomes (chromosome 1: 5, chromosome 2: 3), but not on any plasmid (Figure 1B). Comparing the genomic location with the predicted 5′- and 3′-UTRs [16] reveals that four of the UTR-derived sRNAs are located in 5′-UTRs (UdsA, UdsE, UdsF and UdsG) and four in 3′-UTRs (UdsB, UdsC, UdsD and UdsH). An RT-PCR approach with specific primers for each sRNA and its corresponding mRNA was used to verify that these sRNAs are truly UTR-derived and stem from sRNA-mRNA cotranscripts (Figure S1). According to the Rfam database (version 14.6), none of the 5′ UTR-derived sRNAs is a riboswitch.

Next, a master dataset of sRNAs from *R. sphaeroides* was generated using (a) previously predicted sRNAs (*n* = 50), (b) a list of already described and validated sRNAs (*n* = 23), and (c) novel predicted and validated UTR-derived sRNAs form this study (*n* = 5; general feature file of master dataset see Supplementary Materials). Subsequently, all sRNAs were classified according to their genomic origin using BEDtools window (version 2.25.0, [47]) with predicted 5′/3′ untranslated regions [16]) and the general feature file as input. The majority of sRNAs is classified as orphan (38 of 79 in total), 18% originate from RNA 5′-UTRs and 20% from 3′-UTRs (Figure 1C). 14% of all sRNAs listed in the master database could not properly assigned to any distinct origin.

### 2.2. Global Prediction of UTR-Derived sRNA Generation Mechanisms

**Figure 2:**
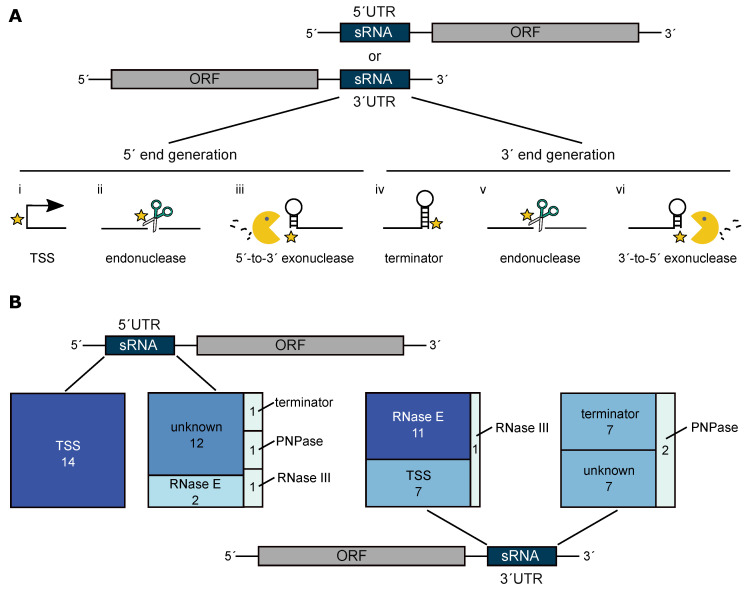
Predicted generation mechanisms of all UTR-derived sRNAs. (**A**) Independently of the location (5′ or 3′ UTR), the sRNA ends can be generated via different mechanisms. The options for the 5′ end generation include a transcription start site (i), an endonucleolytic cleavage (ii) or a 5′-to-3′ processing (iii). A terminator (iv), an endonucleolytic cleavage or an 3′-to-5′ exonuclease can contribute to the RNA 3′ end formation. (**B**) The generation mechanism of all UTR-derived sRNA ends in *R. sphaeroides* was determined using predictions of transcription start sites, Rho-independent terminators and RNase III/RNase E/PNPase-dependent 5′/3′ ends. All 5′ ends of the 5′ UTR-derived sRNAs are generated by transcription start sites, the 3′ ends are mostly formed by an unknown mechanism. RNase E (5′ ends) and Rho-independent terminators (3′ ends) account for the formation and processing of 3′ UTR-derived sRNAs. Nevertheless, unknown factors are likely to contribute to the 3′ end generation.

Several different enzymes and features can be involved in the generation of UTR-derived sRNAs. Independently of the origin (5${}^{\prime }$ or 3${}^{\prime }$ UTR), an sRNA 5${}^{\prime }$ end can be generated by a transcription start site (TSS), an endonucleolytic cleavage or by a 5${}^{\prime }$-to-3${}^{\prime }$ exoribonuclease (Figure 2A). Options for the generation of the sRNA 3${}^{\prime }$ ends include a terminator, endonucleolytic cleavage or pausing of 3${}^{\prime }$-to-5${}^{\prime }$ processing by an exonuclease such as PNPase. To predict those mechanisms on a genome-wide scale, we first searched for overlaps between all UTR-derived sRNAs, and predicted TSS [16] as well as Rho-independent terminators. Second, all RNA 5${}^{\prime }$/3${}^{\prime }$ ends that are dependent on RNase E, RNase III or PNPase were computed with XPEAP (version 1.0.1) as described earlier [37]. Next, all overlaps with these RNA 5${}^{\prime }$/3${}^{\prime }$ ends were computed with BEDtools function window (version 2.25.0; [47]). Every feature that is located within a window of −5 nt to +5 nt of every sRNA 5′ or 3′ end is considered as a potential generation mechanism for this particular RNA end. If more than one feature is assigned to that window, all are considered as generation mechanisms in the downstream analysis, since as a matter of principle even two features which are in close proximity can both contribute to the corresponding RNA end generation. RNA 5′/3′ ends lacking any overlap with the input features were classified as unknown regarding the respective mechanism by which they were generated. The analysis reveals that all 5′ ends of 5′ UTR-derived sRNAs are generated by transcription start sites, whereas the 3′ ends are mainly generated by a so far unknown mechanism (Figure 2B). Nevertheless, RNase E, RNase III and also PNPase and a Rho-independent terminator are each responsible for at least one RNA 3′ end. The picture changes when looking at the 5′ ends of 3′ UTR-derived sRNAs: endonucleolytic cleavage by RNase E is predicted to play a major role and accounts for eleven 5′ ends. The second major part is predicted to be generated by TSS. This finding may be biased, because the TSS prediction performed by Remes et al. [48] is based on a comparison between RNA samples that were treated or untreated with TEX (terminator 5′-phosphate dependent 5′-to-3′ exoribonuclease). In the past, we observed that the transcription start site prediction for sRNAs sometimes resulted in false positive hits which may be linked to the high quantity of sRNAs compared to mRNAs. Furthermore, sRNAs are highly structured, which may protect them from degradation by TEX. The 3′ ends of 3′ UTR-derived sRNAs are predicted to depend mainly on terminator structures and also on PNPase. In total, seven of these 3′ ends could not be assigned to any feature.

### 2.3. Several Enzymes Account for The Maturation and Processing of UTR-Derived sRNAs In Vivo

**Figure 3:**
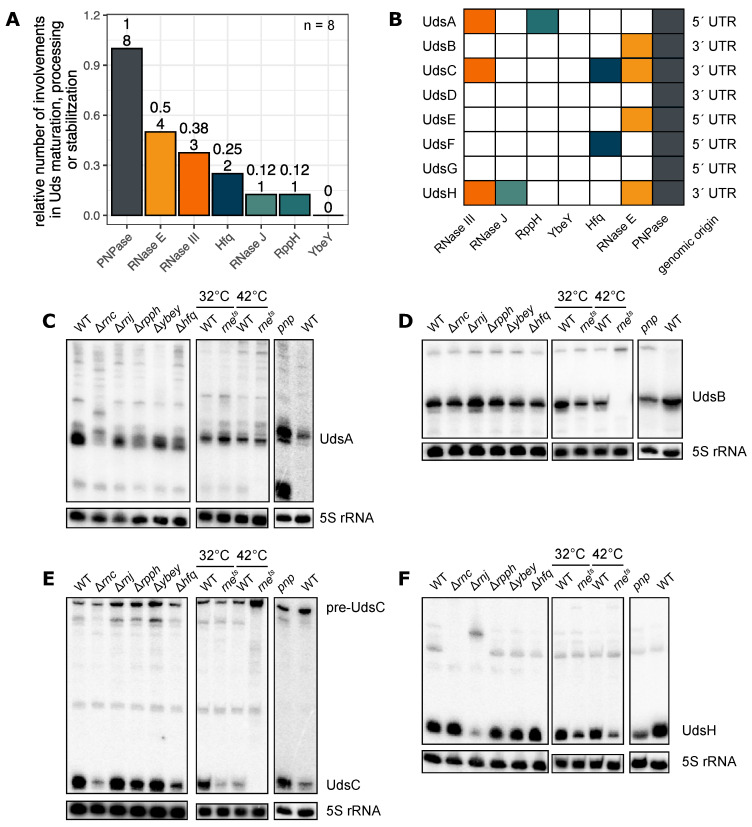
The processing and maturation of UTR-derived sRNAs in *R. sphaeroides* is influenced by various different enzymes. (**A**) Total RNA was isolated from the depicted strains and analyzed by northern blots. If the processing pattern, the abundance of a precursor RNA or the final sRNA varied comparing the wild type and a mutant strain, the respective enzyme was classified as “involved in Uds maturation, processing or stabilization”. *n* = 8. Full blots with samples from biological triplicates are shown in Figures S2–S4. (**B**) Summary of the involved enzymes subdivided by individual UTR-derived sRNAs. Colors represent the enzymes also depicted in Figure 3A. (**C**–**F**) Northern blots illustrating the processing and maturation of UdsA, UdsB, UdsC and UdsH. 10 μg of total RNA per lane. 5S rRNA serves as loading control. Northern blot of *pnp* and wild type in (**C**) was first published by Spanka et al. [37] under Creative Commons Attribution 4.0 International License (https://creativecommons.org/licenses/by/4.0/, accessed on 7 November 2021).

To compare the previously described predictions for the RNA end formation with in vivo data, strains with deletions of the genes coding for RNase III, RNase J, YbeY, RppH and Hfq were used. As RNase E is essential in *Rhodobacter sphaeroides*, a mutant strain was generated using a thermosensitive RNase E from *E. coli* [20]. This enzyme shows a reduced catalytic activity at 32 °C and is even more impeded at 42 °C. In *R. sphaeroides* PNPase is essential too, so the RNA binding domains KH/S1 were removed by insertion of an in-frame stop codon [37]. Total RNA from the mutant strains *pnp*, *rne^E. coli (ts)^*, Δ*rnc*, Δ*rnj*, Δ*ybeY*, Δ*rppH* and Δ*hfq* was isolated from exponentially growing cultures. Next, a northern blot analysis was performed to compare the sRNA levels between the different mutant strains and the wild type. Most remarkably, the 3′-to-5′ exononuclease PNPase is involved in the maturation or processing of all analyzed Uds’ (Figure 3A,B). The mature sRNA levels can either be increased (UdsA, Figure 3C) or decreased (UdsB, Figure 3D). PNPase is known to have an important role in degradation of sRNAs that are associated with Hfq [35], a role in sRNA maturation that, to the best of our knowledge, was not reported. The second enzyme with major impact on maturation/processing of the Uds’ is the endoribonuclease RNase E that influences the maturation of four of the analyzed sRNAs (UdsB, UdsC, UdsE und UdsH). The mature sRNAs UdsB and UdsC can be detected in the *rne^E. coli (ts)^* mutant strain at the permissive temperature (32 °C) but are not detectable at the non-permissive growth temperature of 42 °C. Instead, precursor molecules are strongly enriched in the mutant strain, indicating that RNase E is required for maturation of these sRNAs (Figure 3D,E). RNase E is known to be important for sRNA maturation in *R. sphaeroides* [16], *Vibrio cholerae* [24,25] and *Salmonella enterica* [26]. Moreover, the endonuclease RNase III accounts for the processing of three Uds’ (UdsA, UdsC, UdsH; Figure 3). The RNase J is known to act as 5′-to-3′ exonuclease involved in 23S rRNA maturation in *R. sphaeroides* [38]. It is also involved in the maturation of the sRNA UdsH: precursor RNAs are enriched, whereas the mature sRNA levels are decreased in the Δ*rnj* mutant strain (Figure 3F).

Overall, the predicted generation mechanisms and the enzymes which are involved in the processing reactions in vivo agree in most cases (Table 1). The northern blots for UdsA and UdsG also hint to an involvement of RNase E, the results are, however, not as clear as for other sRNAs (Figures S2A and S4A).

### 2.4. Growth Conditions Impact the UTR-Derived sRNA Levels

#### 2.4.1. Uds’ Abundances Are Growth Phase Dependent

**Figure 4:**
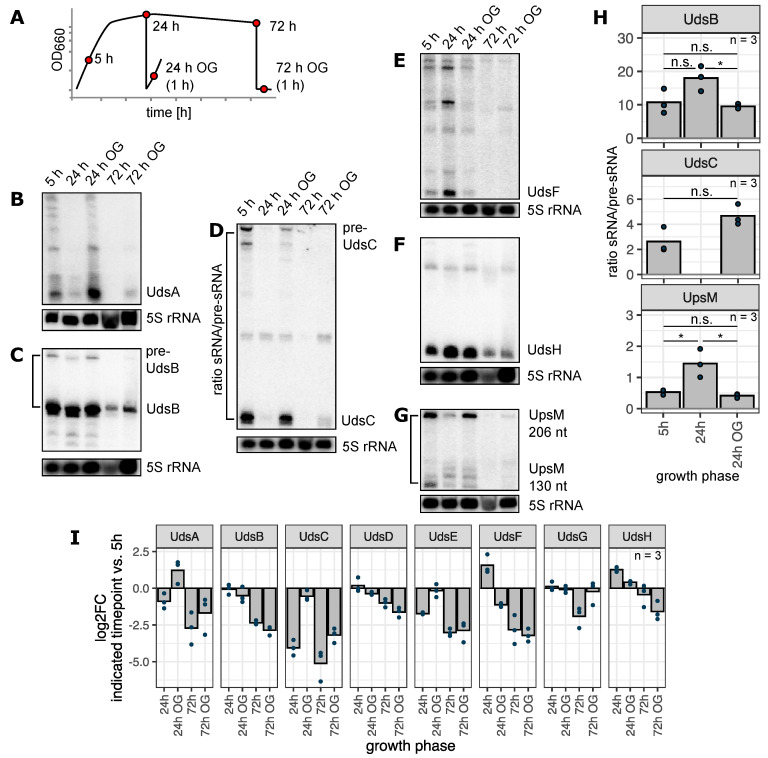
The UTR-derived sRNA levels strongly depend on the growth phase. (**A**) *R. sphaeroides* wild type cultures were incubated for 72 h under microaerobic conditions. Cells were harvested at the depicted times (red dots). After 24 h and 72 h, outgrowth cultures were inoculated. (**B**–**G**) Total RNA from the depicted samples was analyzed by northern blot using specific probes against UdsA to UdsH and UpsM. The sRNA levels vary throughout the different growth phases and can be increased or decreased when compared to the levels during the exponential phase. Loading control: 5S rRNA. Full blots with samples from biological triplicates are shown in Figure S5. Membranes were used with multiple probes: UdsA, UdsD and UdsE; UdsB, UdsG, UdsH and UpsM; UdsC and UdsF. (**H**) Signals of the sRNAs UdsB, UdsC and UpsM and their corresponding pre-sRNAs were quantified and the ratio was calculated (y-axis). Bars indicate the mean value, every dot represents one biological replicate, *n* = 3. Groups were compared with the two-sided Student’s *t*-test: * *p*-value < 0.05; n.s. not significant. (**I**) log_2_fold changes were computed comparing the sRNA abundances during the growth phases with the respective signal in the 5 h sample. Quantification based on northern blot data, *n* = 3.

To analyze the impact of growth phase on maturation of UTR-derived sRNAs, *R. sphaeroides* liquid cultures were incubated for 72 h, and total RNA was isolated during the exponential (5 h), early stationary (24 h) and late stationary phases (72 h). Moreover, outgrowth (OG) cultures were inoculated after 24 h and 72 h and cultivated for 1 h (Figure 4). The Uds sRNA levels were strongly dependent on growth phase: UdsA and UdsC were highly abundant in the exponential phase (5 h after inoculation) and during the outgrowth after 24 h but could hardly be detected in the samples from the stationary phase (Figure 4B,D). Other sRNAs such as UdsF and UdsH are highly enriched during the early stationary phase (log_2_fold change > 1.25; Figure 4E,F). Furthermore, a general trend could be observed in the samples taken during the late stationary phase, when nearly all sRNA levels showed lower abundances compared to the exponential phase (Figure 4I). The sRNAs UdsB, UdsC and UpsM are processed by RNase E (this study and [20]). Since the precursor RNAs can be detected on northern blots, we quantified these signals and calculated the ratio sRNA/pre-sRNA (Figure 4H). Remarkably this ratio increases after 24 h for the sRNAs UdsB and UpsM (two-sided Student’s *t*-test, *p*-value ≤ 0.05), whereas the ratio is not significantly changed comparing the exponential phase and the 24 h outgrowth (two-sided Student’s *t*-test, *p*-value > 0.05). This observation suggests an increased processing of the sRNAs UdsB and UpsM by RNase E during the early stationary phase.

**Figure 5:**
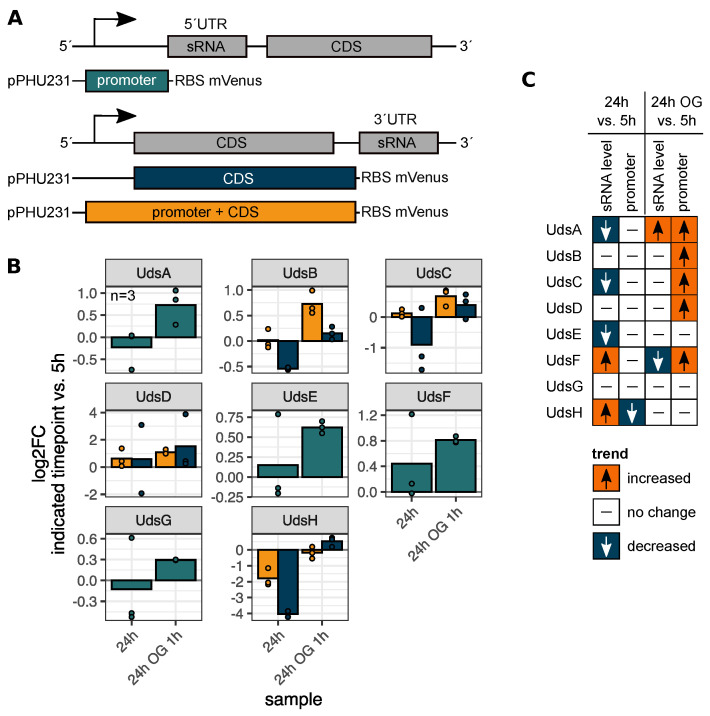
The UTR-derived sRNA promoter activity is growth phase dependent but does not represent the observed sRNA level in all cases. (**A**) Overview of the transcriptional promoter fusions used in this study. (**B**) The plasmids were conjugated in the wild type strain and the fluorescence intensity was measured after 5 h, 24 h and from the outgrowth culture. x-axis: sample. y-axis: log_2_fold change of the indicated timepoint vs. signal during exponential growth phase (5 h). Green: promoter sequence. Blue: coding sequence (CDS). Yellow: promoter + coding sequence. *n* = 3. Every dot represents the mean value of two technical replicates. Signals F/OD_660_ are shown in Figure S6. (**C**) Classification of the sRNA level and the promoter activity, based on northern blot data and fluorescence intensities. Red: increased compared to the exponential phase. Blue: decreased compared to the exponential phase. White with horizontal dash: no change.

To investigate the influence of an altered promoter activity on the sRNA levels, transcriptional promoter fusions were constructed. For the 5${}^{\prime }$ Uds’, only the promoter sequence was fused to mVenus. In contrast to that, two sequences were used for each of the 3${}^{\prime }$ Uds’: the upstream coding sequence (CDS) and a longer fragment containing the promoter of the upstream gene and the CDS (Figure 5A,B). This strategy allows the detection of putative internal promoters in case of 3${}^{\prime }$ UTR-derived sRNAs. According to the upstream sequences, the promoters of *udsA* and *udsH* depend on the alternative sigma factors RpoH_I_/RpoH_II_ and the promoter of *RSP_7527-udsC* is RpoH_II_ dependent (Table S1). Promoter sequences of the other UTR-derived sRNAs do not accord with the published RpoH_I_/RpoH_II_ or RpoH_II_ consensus motifs [49]. The growth experiment was repeated with wild type strains harbouring the described plasmids. To avoid misleading results caused by the high protein stability followed by an accumulation of mVenus, samples were only taken after 5 h and 24 h of cultivation and from the outgrowth cultures. The normalized fluorescence intensities varied substantially among the different promoter constructs, ranging from an F/OD of 100 (UdsH) to 4000 (UdsB) after 5 h of cultivation (Figure S6). For those two sRNAs in particular, the constructs harbouring only the CDS exhibited a detectable fluorescence signal, which was nevertheless lower than the signal from the promoter + CDS constructs (Figure S6). This indicates a transcription of the sRNA by two promoters, one belonging to the cotranscribed gene and one located in the open reading frame. Except for the UdsG promoter, the activity of all other promoters was increased or decreased when comparing the early stationary phase and respective outgrowth to the exponential phase. The 3${}^{\prime }$ derived UdsC and UdsD are exclusively transcribed by the promoter of the upstream gene, while for UdsB and UdsH additional promoter activity within the upstream coding region was detected. 

Next, we compared the observed sRNA levels and corresponding promoter activities using a trend heatmap (Figure 5C). For every sRNA and promoter construct the relative change in signal intensity comparing the 24 h and outgrowth sample to the exponential phase was computed. Samples with a log_2_fold change > 0.65 were classified as “increased”, log_2_fold change < −0.65 as “decreased” and all others as “no change”. In case of UdsA the changes of sRNA level and promoter activity show the same trend in the 24 h outgrowth cultures. For all other sRNAs the changes of sRNA levels cannot be solely due to changed promoter activity. This points to an important role of sRNA maturation in the growth phase dependent expression of sRNAs.

#### 2.4.2. External Stressors Affect the sRNA Abundances

**Figure 6:**
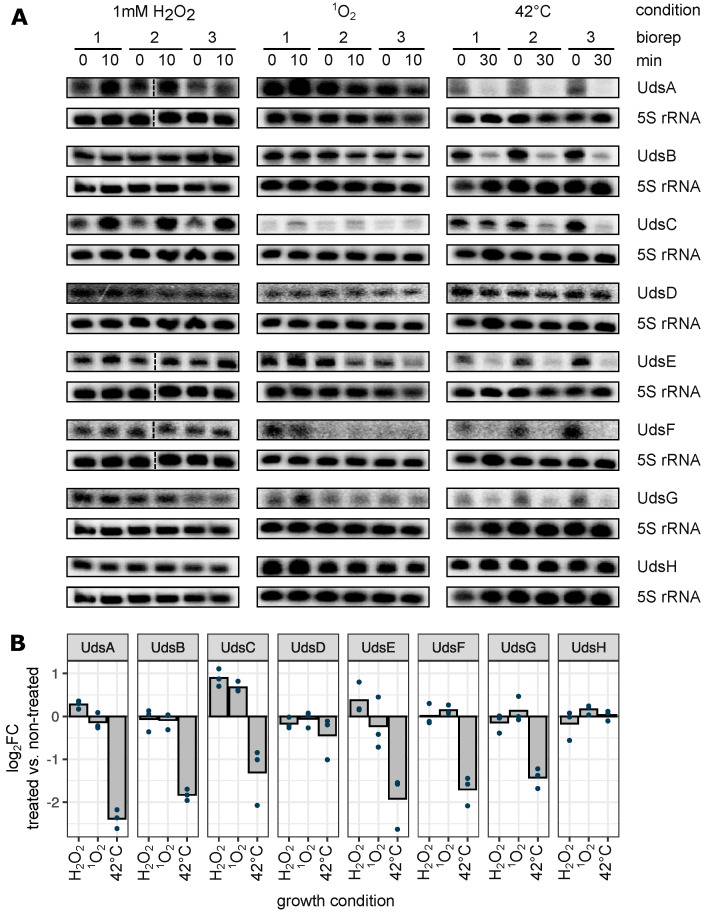
UTR-derived sRNAs are influenced by various stress conditions. (**A**) Exponentially growing *R. sphaeroides* liquid cultures were exposed to oxidative (1 mM H_2_O_2_, 10 min), singlet oxygen (^1^O_2_, 10 min) or heat stress (42 °C, 30 min). Samples for RNA isolation were harvested before and after the indicated time. Northern blot analysis of biological triplicates, 10 μg total RNA per lane. 5S rRNA served as loading control. Uncut northern blots are shown in Figures S7 and S8. (**B**) log_2_fold changes (treated vs. non-treated) were computed based on the northern blot data. Every dot represents one biological replicate. *n* = 3.

Previous studies highlighted the important role of sRNAs during the oxidative stress response in *R. sphaeroides* (e.g., [46,50,51]). We asked whether stress conditions also affect the levels of UTR-derived sRNAs by influencing their maturation. Wild type cultures were incubated with 1 mM H_2_O_2_, grown in ^1^O_2_ generating conditions or exposed to a heat shock at 42 °C. RNA samples were taken before and after the treatment and subsequentially analyzed via northern blot (Figure 6). The abundances of nearly all Uds’ are influenced by at least one external stressor; only UdsD and UdsH showed a more or less stable signal independently of the growth condition (Figure 6A). The sRNA UdsC showed a strong dependence on oxidative stress that was induced by hydrogen peroxide and singlet oxygen (mean log_2_fold change > 0.65, Figure 6B). A general trend could be observed after heat shock induction, since all sRNA abundances were reduced except of the UdsD and UdsH levels.

**Figure 7:**
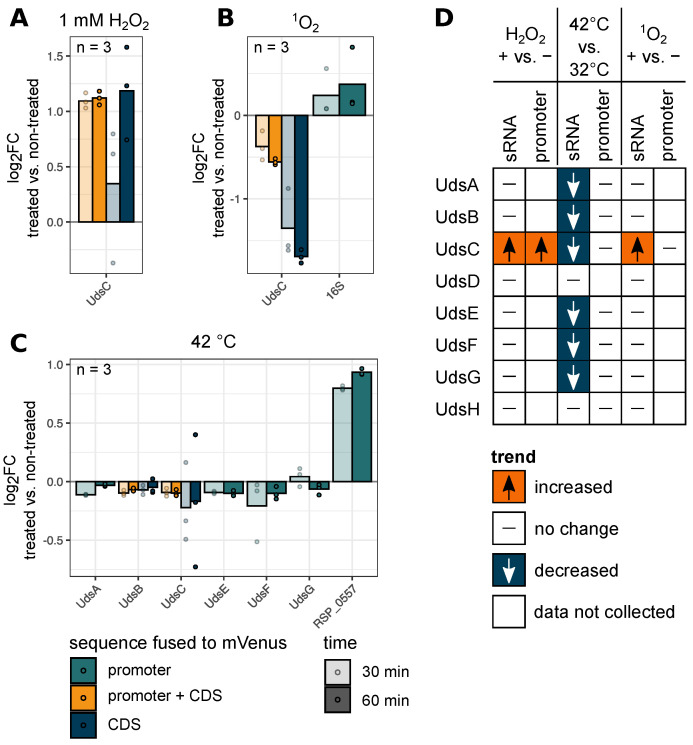
Oxidative and heat stress have a major impact on several UTR-derived sRNA promoter activities. Fluorescence intensity of the wild type harbouring the indicated plasmids was measured before and after treatment with 1 mM H_2_O_2_ (**A**), ^1^O_2_ stress (**B**), 42 °C heat stress (**C**) and the log_2_fold changes were calculated. x-axis: sample. y-axis: log_2_fold change treated vs. non-treated. Green: promoter sequence. Blue: coding sequence (CDS). Yellow: promoter + coding sequence. Color intensity indicates duration of induction. *n* = 3. Every dot represents the mean value of two technical replicates. The promoters of *RSP_0557* (unpublished data) and 16S rRNA (McIntosh et al., 2019) were used as positive controls for the indicated growth conditions. Signals F/OD_660_ are shown in Figure S9F. (**D**) Classification of the sRNA level and the promoter activity, based on northern blot data and fluorescence intensities. Red: increased compared to non-treated sample. White with horizontal dash: no change. Blue: decreased compared to non-treated sample. White without dash: data not collected.

All promoter activities under stress conditions were tested for those sRNAs which exhibited log_2_fold changes > 0.65 or < −0.65 on the northern blot under stress conditions (eight combinations of stress condition and construct in total, Figure 7). We only observed an increased fluorescence intensity for the promoter construct of UdsC under H_2_O_2_ stress and a decreased signal of the UdsC promoter during singlet oxygen stress (Figure 7A,B). In contrast to that, a shift to 42 °C led mainly to constant signals and only the strain harbouring the promoter fusion of *RSP_0557* (positive control) showed an increasing fluorescence intensity over time (Figure 7C). Next, the change in fluorescence signal was categorized to visualize the trends as described above (Figure 7D). Similar to the analyzed growth experiments, only one comparison showed the same trend between the sRNA levels and the corresponding promoter activities. These results strongly suggest that UTR-derived sRNA levels rely on transcription rate and factors like processing events or altered degradation rates which contribute to the mature sRNA abundances. Nevertheless, no specific precursor RNAs or enriched degradation products could be found that might be linked to the function of one specific ribonuclease responsible for that particular processing reaction.

**Figure 8:**
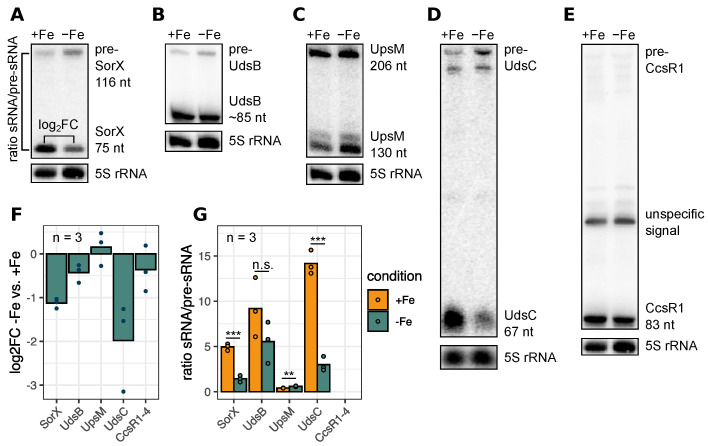
Iron availability influences the RNase E dependent processing of UTR-derived sRNAs. Total RNA from *R. sphaeroides* wild type cultures grown in media supplemented with iron (+Fe) or after iron depletion (−Fe) was isolated and analyzed via northern blot. Membranes were hybridized with probes directed against sRNAs which are processed by RNase E: SorX (**A**), UdsB (**B**), UpsM (**C**), UdsC (**D**) and CcsR1 (**E**). The experiment was performed in biological triplicates. 5S rRNA served as loading control. Full blots with samples from biological triplicates are shown in Figure S10. (**F**) log_2_fold changes of the mature sRNA species were calculated comparing the −Fe and +Fe conditions. Grey bars indicate the mean value, every dot represents one biological replicate, *n* = 3. (**G**) Signals of the sRNAs and pre-sRNAs were quantified and the ratio was calculated (y-axis). Bars indicate the mean value (+Fe: yellow, −Fe: green), every dot represents one biological replicate, *n* = 3. Groups were compared with the two-sided Student’s *t*-test: *** *p*-value < 0.001; ** *p*-value < 0.01; n.s. not significant. Signal of the CcsR1 precursor was too low for quantification.

Analyzing RNA-Seq data from *R. sphaeroides* grown under iron limitation revealed that the RNase E generated UdsB was slightly less abundant compared to the control grown in media with supplemented iron. However, the *RSP_1771* part of the *RSP_1771-udsB* cotranscript was more abundant. This prompted us to investigate if the RNase E mediated processing of UTR-derived sRNAs is influenced by iron availability during the exponential growth phase. Total RNA was isolated from *R. sphaeroides* cultures grown in malate minimal media supplemented with iron or under iron depleted conditions [52] and analyzed via northern blot. RNase E is involved in maturation of SorX, UpsM, CcsR1-4, UdsB and UdsC from precursors ([15,17,20]; this study). We therefore quantified the mature sRNA levels and, if possible, the respective precursor RNAs from northern blots (Figure 8A–E). Next, the log_2_fold changes between iron replete and iron deplete conditions and the signal ratios were computed (Figure 8F,G). We observed that the ratio of sRNA to precursor sRNA is significantly reduced for SorX and UdsC when the cultures were grown in iron depleted medium, indicating a reduced processing rate by RNase E for these sRNAs (two-sided Student’s *t*-test, *p*-value < 0.001). In contrast to that, the ratio of UdsB/pre-UdsB was not significantly decreased, and in the case of UpsM, the ratio even increased (Figure 8C,G). Our results indicate that RNase E processing of UTR-derived sRNAs is modulated under iron limiting growth conditions in a substrate dependent manner.

## 3. Discussion

Our data demonstrate that the maturation of sRNAs from UTRs is an important step for the control of sRNA levels. As a result, levels of the co-transcribed mRNA and sRNA can respond differently to environmental changes. Why may such differential regulation be appropriate? To address this question, it is important to know the function of the mRNA and the function of the sRNA, which is unfortunately the case for only a few examples. Transcription of mRNA and the UTR-derived sRNA from the same promoter leads to the production of similar levels of both, and to the same transcriptional regulation. This seems reasonable if both RNAs have a function in the same pathway and/or affect the same physiological process as already shown for some UTR-derived sRNAs. e.g., CpxQ is derived from the 3${}^{\prime }$ UTR of *cpxP* and both RNAs are involved in the inner membrane stress response in *Salmonella enterica* [26]. In the same bacterium, NarS is derived from the 3${}^{\prime }$ UTR of *narK* that encodes a nitrate transporter. NarS is involved in the cross-regulation of nitrate and nitrite transport [53]. In enterohemorrhagic *E. coli*, StxS is derived from the 5${}^{\prime }$ UTR of *stx1AB* for Shiga toxin 1 by premature transcriptional termination. StxS represses Shiga toxin 1 production under lysogenic conditions [54]. In *Pseudomonas aeruginosarhII* encodes the enzyme for AHL synthesis. RhlS is derived from its 5${}^{\prime }$ UTR of *rhII* and is required for the production of normal levels of AHL [55]. In *E. coli*, MalH is derived from the 3${}^{\prime }$ UTR of the maltose uptake operon *malEFG* and contributes to alternative carbon source utilization by affecting maltoporin expression [56]. The product of *argR* and the 3${}^{\prime }$ UTR-derived ArgX, both regulate the arginine deiminase pathway in *Lactococcus lactis* [57]. Some UTR-derived sRNAs were also characterized in *R. sphaeroides*: the 3${}^{\prime }$ UTR-derived SorX and the *ompR1* mRNA, both function in the oxidative stress response [15,58]. The 3${}^{\prime }$ UTR-derived PcrX RNA and the *puf* operon are required for formation of photosynthetic complexes and their regulation [19]. The CcsR1-4 RNAs are derived from the 3${}^{\prime }$ UTR of *ccaF1* (*RSP_6037*), which encodes a small RNA-binding protein required for the maturation of CcsR and other sRNAs [17,18]. It seems reasonable that all these sRNAs are under the control of the same promoter as the related mRNA. Why have another level of regulation at the step of sRNA maturation?

Many bacterial genes are organized in polycistronic operons and consequently regulated by the same promoter. Nevertheless, an additional regulation at the posttranscriptional level can result in a different abundance of mRNA segments that determine the stoichiometry of the resulting proteins. In case the of e.g., the *puf* operon of *R. capsulatus*, this is due to segmental differences in mRNA stability [59]. Differences in initiation of translation for individual genes of an operon were demonstrated e.g., for the *atp* operon [60] or the *gal* operon [61] in *E. coli*. Partial transcriptional termination leads to differential expression of the genes in e.g., the *E. coli rpsO-pnp* operon [62]. It is conceivable that in case of UTR-derived sRNAs the change of the ratio of the sRNA and mRNA is favorable under certain environmental conditions, but this needs to be tested in the future.

Although our data demonstrate that environmental factors can influence maturation of UTR-derived sRNA and also point to some of the mechanisms involved in the maturation of the individual sRNAs, the exact mechanisms underlying the regulation need further investigation. If a second promoter is contributing to the generation of a 3${}^{\prime }$ UTR-derived sRNA, this may of course lead to an expression pattern that is different from that of the mRNA. But transcriptional regulation may also account for different levels of RNases or indirectly affect the level of an UTR-derived sRNA. In case of the *R. sphaeroides* sRNA UpsM, growth phase-dependent levels are mediated by base pairing to another sRNA, StsR [21]. UpsM is derived from the 5${}^{\prime }$ UTR of the *dcw* (division and cell wall synthesis) gene cluster in *R. sphaeroides* by partial transcriptional termination [20]. The orphan sRNA StsR is induced during the stationary growth phase by the alternative sigma factors RpoH_I/II_ [48]. StsR base pairs to UpsM and the 5${}^{\prime }$ UTR of the *dcw* genes, resulting in a structural change which gives access to an RNase E cleavage site within the *upsM* sequence. Interestingly, the interaction to StsR and the subsequent cleavage of the *dcw* 5${}^{\prime }$ UTR also affects read through into the *dcw* genes [21].

**Figure 9:**
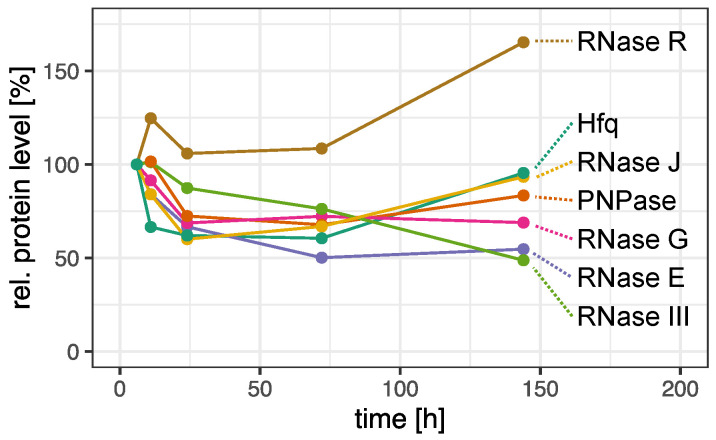
The levels of several RNases and of the RNA chaperon Hfq vary at different stages of growth as determined by quantitative mass spectrometry. *R. sphaeroides* wild type cultures were grown under microaerobic conditions and sampled in mid exponential phase (OD_660_ = 0.5), in transition to stationary phase (11 h after inoculation, OD_660_ ≈ 1.0), in early stationary phase (24 h after inoculation, OD_660_ ≈ 1.8) and in late stationary phase (72 and 144 after inoculation, OD_660_ ≈ 1.1) and a quantitative proteome analysis was performed as described in Bathke et al. [63]. Values for the RNases and for Hfq are taken from the data set of this publication.

Furthermore, the amount or activity of the RNases may be altered in response to environmental cues. Changing amounts of RNases can be due to transcriptional regulation or proteolysis. Quantitative mass spectrometry data from a previous study [63] revealed that the protein levels of several RNases vary through the different growth phases, which includes significant changes in RNase E, III, P, PNPase and also Hfq levels (Figure 9). This may influence both the processing and stability of UTR-derived sRNAs and thus contribute to a modulation of sRNA level dependent on the growth phase. Moreover, post-translational modifications can alter the stability of RNases (reviewed in [64]). e.g., higher levels of RNase R were reported under stress or in stationary phase in *E. coli* [65] and attributed to stress-dependent reduction of acetylation [66]. The activity of RNases can be affected by post-transcriptional modifications or by cellular localization (reviewed in [64]). This might also be the case for RNase E: We observed an increased RNase E mediated processing of two UTR-derived sRNAs during the early stationary growth phase (Figure 4), although the protein level was about 35% reduced (Figure 9) and the promoter activities of these UTR-derived sRNAs remained constant (Figure 5). In case of heat stress, it is also conceivable that changes in RNA structure can lead to altered maturation. Our study reported an influence of iron depletion in the maturation of several sRNAs by RNase E. However, the RNase E dependent maturation of other sRNAs was not influenced by iron availability, excluding a general effect of iron availability on RNase E activity.

Most likely, it will not be possible to address the exact mechanisms underlying regulated sRNA maturation at a global scale. A better understanding of the importance and the role of UTR-derived RNAs will need a closer look at the maturation processes and their regulation in the future.

## 4. Material and Methods

### 4.1. Bacterial Strains and Growth Conditions

The strains used in this study are described in Table S2. Erlenmeyer flasks with a volume of 50 mL were filled with 40 mL of malate minimal media. Microaerobic *Rhodobacter sphaeroides* (recently renamed *Cereibacter sphaeroides* [67]) cultures were incubated at 32 °C under continuous shaking in the dark, resulting in a dissolved oxygen concentration of 25 μM to 30 μM [68]. To apply organic peroxide stress, H_2_O_2_ (1 mM final concentration) was added to the liquid cultures. Aerobic cultivation with induction of photooxidative stress was performed as described by Glaeser and Klug [69]. Briefly, microaerobic liquid cultures were shifted to aerobic growth conditions (approximately 180 μM). They were cultivated in the dark at 32 °C in air-gassed Meplat flasks. Methylen blue acts as a photosensetizer and was added in a final concentration of 0.2 μM. During the exponential growth phase, the cultures were exposed to white light (800 W m^2^) to induce the generation of ^1^O_2_. For the heat shock experiments, pre- and main cultures were incubated at 32 °C in the dark under microaerobic conditions. During the exponential growth phase, cultures were shifted to a 42 °C preheated water bath where they were incubated for 30 min under continuous shaking. To generate iron limitation, cultures were treated as described in Remes et al. [68]. Cultures were grown in medium without supplemented iron with 2,2${}^{\prime }$ dipyridyl (30 μ M, Merck) for three times. In the last pre-culture and in the experimental culture, no 2,2${}^{\prime }$ dipyridyl was added.

### 4.2. Construction of a rppH and a ybeY Deletion Strain

Deletion of the gene *rppH* (*RSP_0931*) in the *Rhodobacter sphaeroides* 2.4.1 wild type strain [70] was carried out by homologous recombination and insertion of a kanamycin resistance gene. The up and down fragments were amplified by PCR using the primer pairs KO_RSP0931_up_f /KO_RSP0931_up_r and KO_RSP0931_dw_f/KO_RSP0931_dw_r. Both fragments were cloned in pPHU281 with EcoRI/BamHI and BamHI/HindIII. The kanamycin resistance gene was inserted between the fragments with BamHI. The plasmid was transformed to *E. coli* S17-1 and then transferred to *Rhodobacter sphaeroides* 2.4.1 by diparental conjugation. Positive clones were selected on malate minimal agar containing 25 μg mL^−1^ kanamycin.

The same procedure was also applied to delete the gene *ybeY* (*RSP_3598*) in the *Rhodobacter sphaeroides* 2.4.1 wild type strain. For the up and down fragment amplification the primer pairs KO_3598_ybeY_up_f/KO_3598_ybeY_up_r and KO_3598_ybeY_dw_f /KO_3598_ybeY_dw_r were used. Plasmid construction was carried out as described above but with a gentamicin resistance gene instead (taken from pPHU45Ω). Clones were selected on malate minimal agar containing 10 μg mL^−1^ gentamicin.

### 4.3. Promoter Activity Assay

DNA fragments harbouring the putative promoter sequences of every UTR-derived sRNA were amplified and fused to the mVenus gene using plasmid pPHU231 as described by Charoenpanich et al. [71] and McIntosh et al. [72]. Restriction enzyme cleavage sites (HindIII/XbaI) were incorporated via the primer sequences as well as a strong ribosome binding site (AGGGGAGAAG). Final plasmids were conjugated to the *Rhodobacter sphaeroides* wild type using the *E. coli* S17-1 strain. Liquid cultures were incubated as described above and prediluted to an OD_660_ of 0.15. Volumes of 100 μL liquid culture were transferred to transparent 96-well plates and fluorescence was subsequently measured in the Tecan Infinite M Nano (Tecan Group AG). Primer sequences and cloned constructs are provided in Tables S3 and S4.

### 4.4. Reverse Transcription (RT) PCR

RT-PCR was performed using the Brilliant III Ultra-Fast SYBR Green QRT-PCR Master Mix (Agilent #600886) according to the manufacturer’s manual. DNA free total RNA extracted from exponentially growing wildtype cultures served as template for the reaction. The RT-PCR products were separated on 10% polyacrylamide gels and visualized with ethidium bromide staining.

### 4.5. Northern Blot Analysis

Total RNA was isolated with the hot phenol method [73]. DNase treatment was performed according to the manufacturer’s instructions (Invitrogen #AM1907). The electrophoretic separation on denaturing PAA urea gels was conducted as described by Berghoff et al. [46]. The oligonucleotides were end-labeled using T4 polynucleotide kinase (T4-PNK, Thermo Scientific, Waltham, MA, USA) with [γ^32^P]-ATP (SRP-301, Hartmann Analytic) according to the manufacturer’s protocol. Oligonucleotides used in this study are listed in Table S4. The membranes were washed in 5x SSC buffer after overnight incubation with the labeled oligonucleotides. Sealed membranes were then exposed to a screen for 48 h. The QuantityOne 1-D Analysis Software (BioRad, version 4.6.6) was used to quantify the signals. All sRNA signals were normalized to the 5S rRNA signal which was used as a loading control.

### 4.6. Bioinformatical Analysis

All differential RNA 5${}^{\prime }$ and 3${}^{\prime }$ ends which are RNase E-, RNase III- or PNPase-dependent were identified with XPEAP as described earlier [37]. Parameters used for the computation were: log_2_fold change cutoff ≤−1 or ≥+1; adjusted *p*-value ≤ 0.05 (Benjamini-Hochberg algorithm). Rho-independent transcription terminator prediction was performed using TransTherm HP [74]. Prediction of 5${}^{\prime }$/3${}^{\prime }$ UTRs and TSS was carried out by Remes et al. [48] based on differential NGS RNA-Seq data from *Rhodobacter sphaeroides* 2.4.1. All known and predicted sRNAs in *Rhodobacter sphaeroides* 2.4.1 were first classified according to their genomic origin. Overlaps between annotated sRNAs and predicted 5${}^{\prime }$/3${}^{\prime }$ UTRs were computed with BEDtools window (version 2.25.0, options -s -wa -wb; [47]). Further, for every annotated sRNA all overlaps with (a) predicted transcription start sites (TSS), (b) Rho-independent transcription terminators and (c) differential RNA 5${}^{\prime }$/3${}^{\prime }$ ends that depend on RNase E, RNase III or PNPase were identified using the same function with a window size of 5 nt (10 nt for Rho-independent terminators). Next, windows from position −5 nt to +5 nt at 5${}^{\prime }$ and 3${}^{\prime }$ ends of all sRNAs were defined. All overlapping features that could be assigned to these windows were considered as the putative generation mechanism of this particular sRNA. Multiple overlaps per site were allowed. The read data of all described mutant strains are deposited on NCBI Gene Expression Omnibus: PNPase and RNase III mutant strains (NCBI GEO accession number: GSE156818) and thermosensitive RNase E mutant strain (NCBI GEO accession number: GSE71844, published in Förstner et al. [16]).

## Figures and Tables

**Table 1:** Comparison of the predicted generation mechanisms for the described UTR-derived sRNAs and the determined enzymes, which are involved in processing and maturation in vivo.

sRNA	Origin	Predicted 5${}^{\prime }$ End	Predicted 3${}^{\prime }$ End	Involvement of RNases/Hfq In Vivo
UdsA	5${}^{\prime }$ UTR	TSS	RNase E	RNase III, RppH, PNPase
UdsB	3${}^{\prime }$ UTR	RNase E	terminator	RNase E, PNPase
UdsC	3${}^{\prime }$ UTR	RNase E	terminator	RNase III, RNase E, PNPase, Hfq
UdsD	3${}^{\prime }$ UTR	RNase E	terminator/PNPase	PNPase
UdsE	5${}^{\prime }$ UTR	TSS	unknown	RNase E, PNPase
UdsF	5${}^{\prime }$ UTR	TSS	unknown	Hfq, PNPase
UdsG	5${}^{\prime }$ UTR	TSS	RNase E	PNPase
UdsH	3${}^{\prime }$ UTR	RNase E	terminator	RNase III, RNase J, RNase E, PNPase

## Data Availability

All RNA sequencing data is published at the NCBI Gene Expression Omnibus: PNPase and RNase III mutant strains (NCBI GEO accession number: GSE156818) and thermosensitive RNase E mutant strain (NCBI GEO accession number: GSE71844, published in Förstner et al. [16]).

## Supplementary Materials

The following are available online at https://www.mdpi.com/article/10.3390/ijms222212260/s1.

## Abbreviations

The following abbreviations are used in this manuscript:

CDS	coding sequence
OD	optical density
OG	outgrowth
RT	reverse transcription
TEX	terminator 5${}^{\prime }$-phosphate dependent 5${}^{\prime }$-to-3${}^{\prime }$ exoribonuclease
TSS	transcription start site
UTR	untranslated region
Uds	UTR-derived sRNA
