# Supplementary material for: Maturation of UTR-Derived sRNAs Is Modulated during Adaptation to Different Growth Conditions
Source: Int J Mol Sci. 2021 Nov 12;22(22):12260. doi: 10.3390/ijms222212260 (PMC8625941; doi:10.3390/ijms222212260)
Supplement: Supplementary file 1 [file ijms-22-12260-s001.zip › Supplementary_Figures_and_Tables.pdf]

Supplementary Materials

Maturation of UTR-derived sRNAs is modulated during adaptation to different growth conditions

Daniel-Timon Spanka and Gabriele Klug*

Institute of Microbiology and Molecular Biology, Justus Liebig University Giessen, IFZ, Germany

*: corresponding author, gabriele.klug@mikro.bio.uni-giessen.de

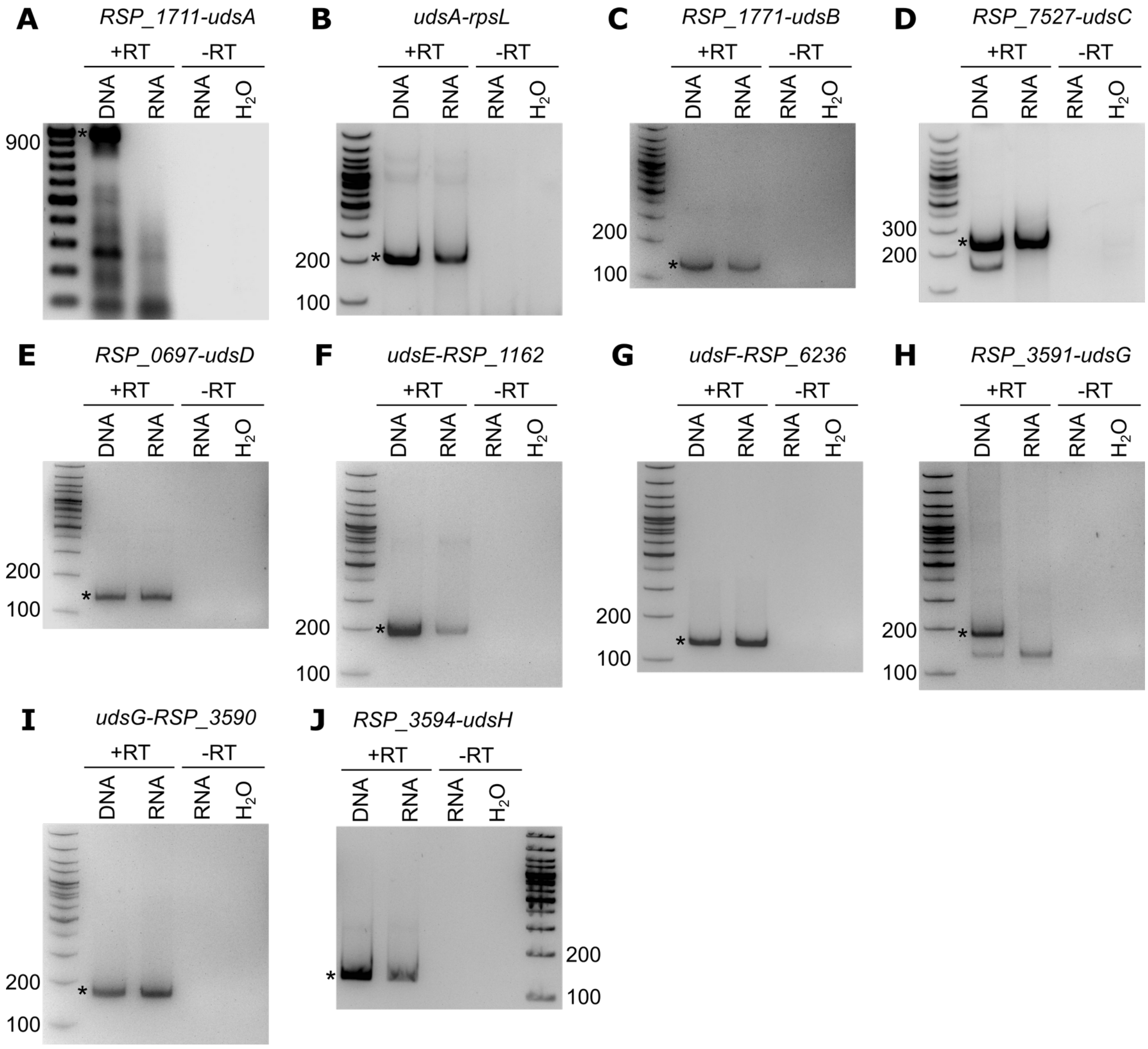

Figure S1. The novel sRNAs are all UTR-derived and cotranscribed with mRNAs. A-J Using RT-PCR, a part of the sRNA and the upstream or downstream located mRNA was amplified and separated on a 10 % PAA gel. UdsA and UdsG are located between two genes which are both in the same orientation, this is why the cotranscription was checked in both directions. UdsA and UdsG are both 5´ UTR derived. All other sRNAs lie only next to one gene. Expected fragment sizes are marked with an asterisk (*).

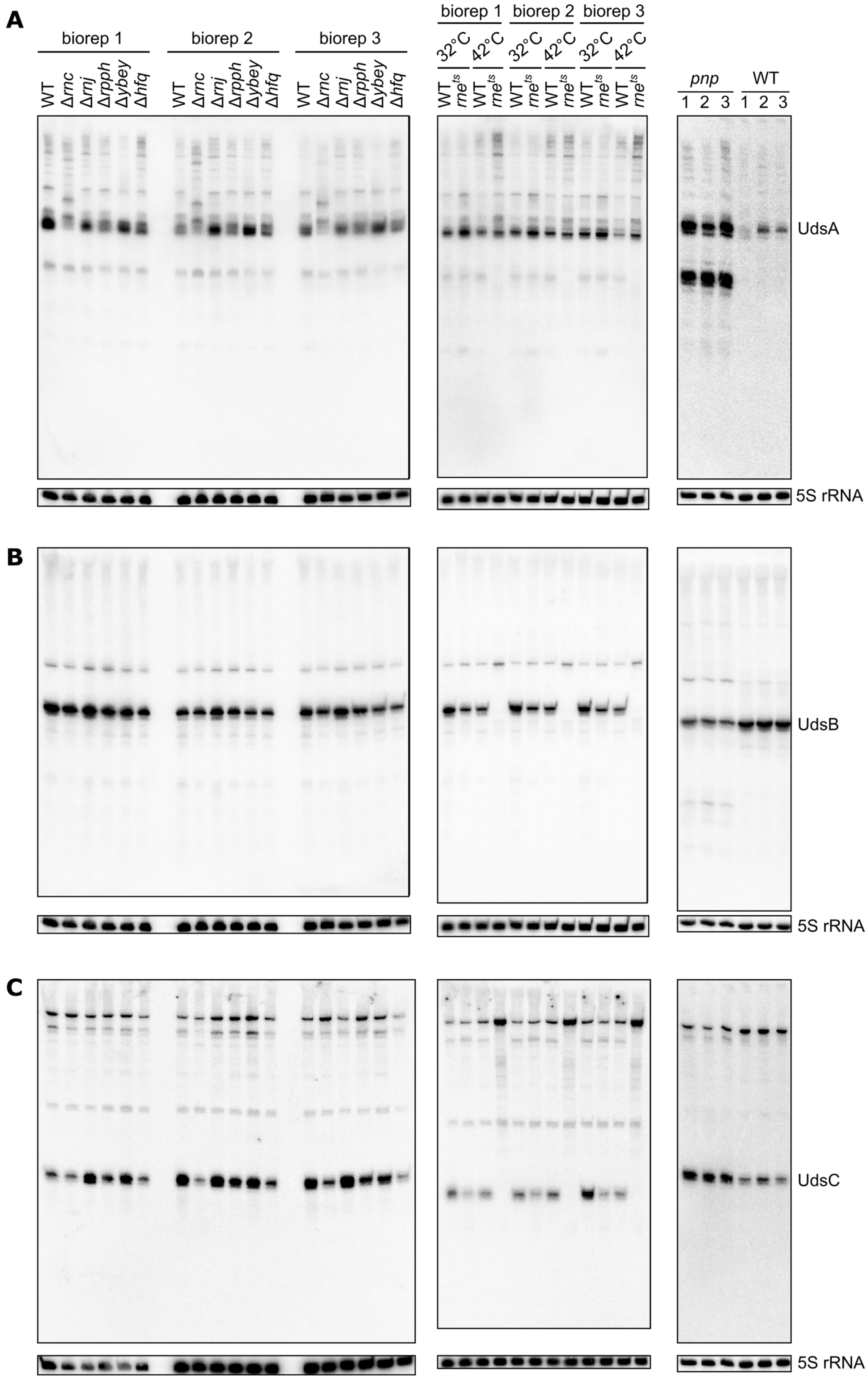

Figure S2. Processing and maturation of UdsA, UdsB and UdsC. During the exponential growth phase, total RNA was isolated from the depicted *R. sphaeroides* strains. 10 µg of total RNA were separated on a denaturating 10 % PAA gel and subsequentially blotted. Membranes were incubated with specific probes against UdsA (**A**), UdsB (**B**) and UdsC (**C**).

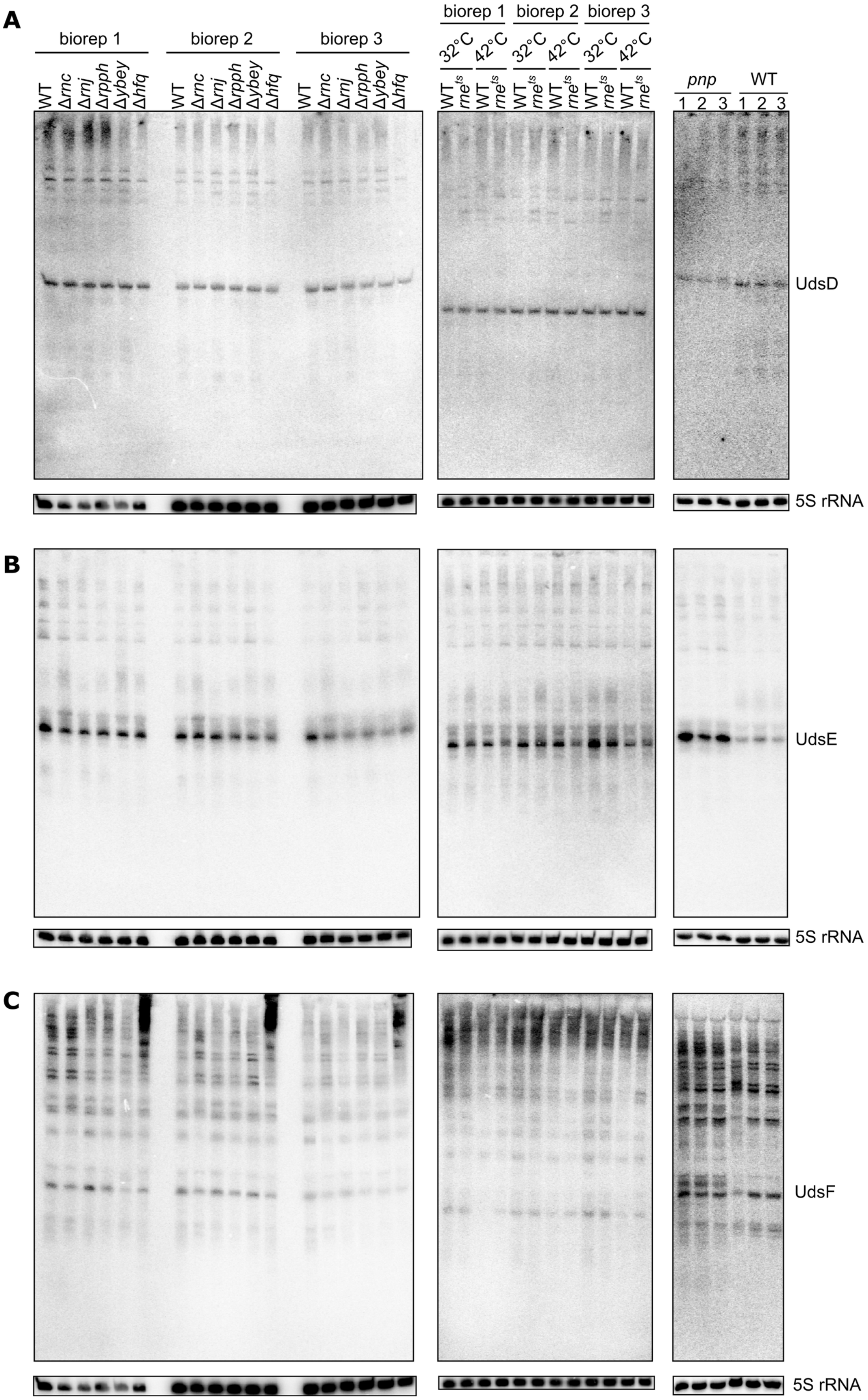

Figure S3. Processing and maturation of UdsD, UdsE and UdsF. During the exponential growth phase, total RNA was isolated from the depicted *R. sphaeroides* strains. 10 µg of total RNA were separated on a denaturating 10 % PAA gel and subsequentially blotted. Membranes were incubated with specific probes against UdsD (**A**), UdsE (**B**) and UdsF (**C**).

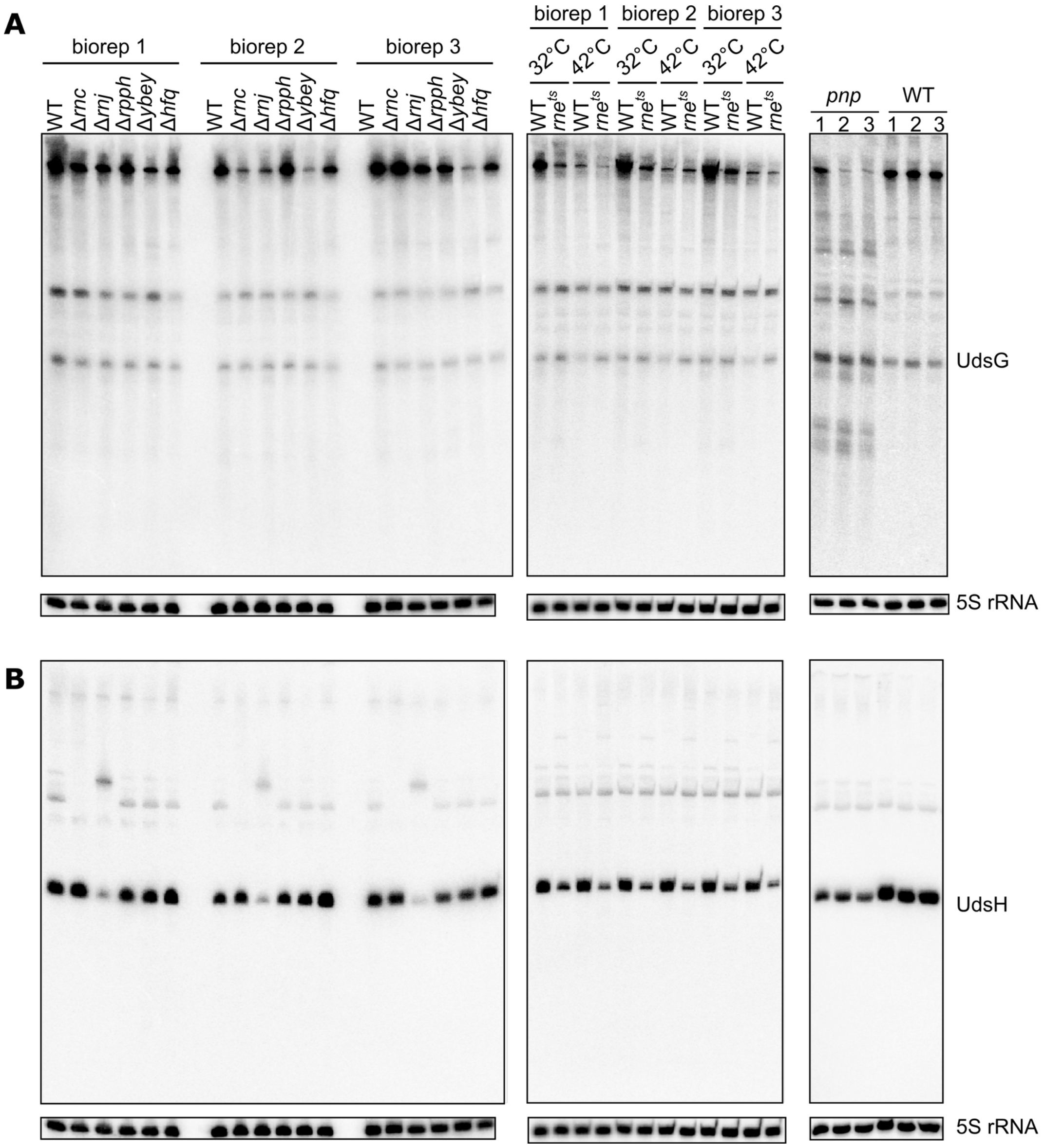

Figure S4. Processing and maturation of UdsG and UdsH. During the exponential growth phase, total RNA was isolated from the depicted *R. sphaeroides* strains. 10 µg of total RNA were separated on a denaturating 10 % PAA gel and subsequentially blotted. Membranes were incubated with specific probes against UdsG (**A**) and UdsH (**B**).

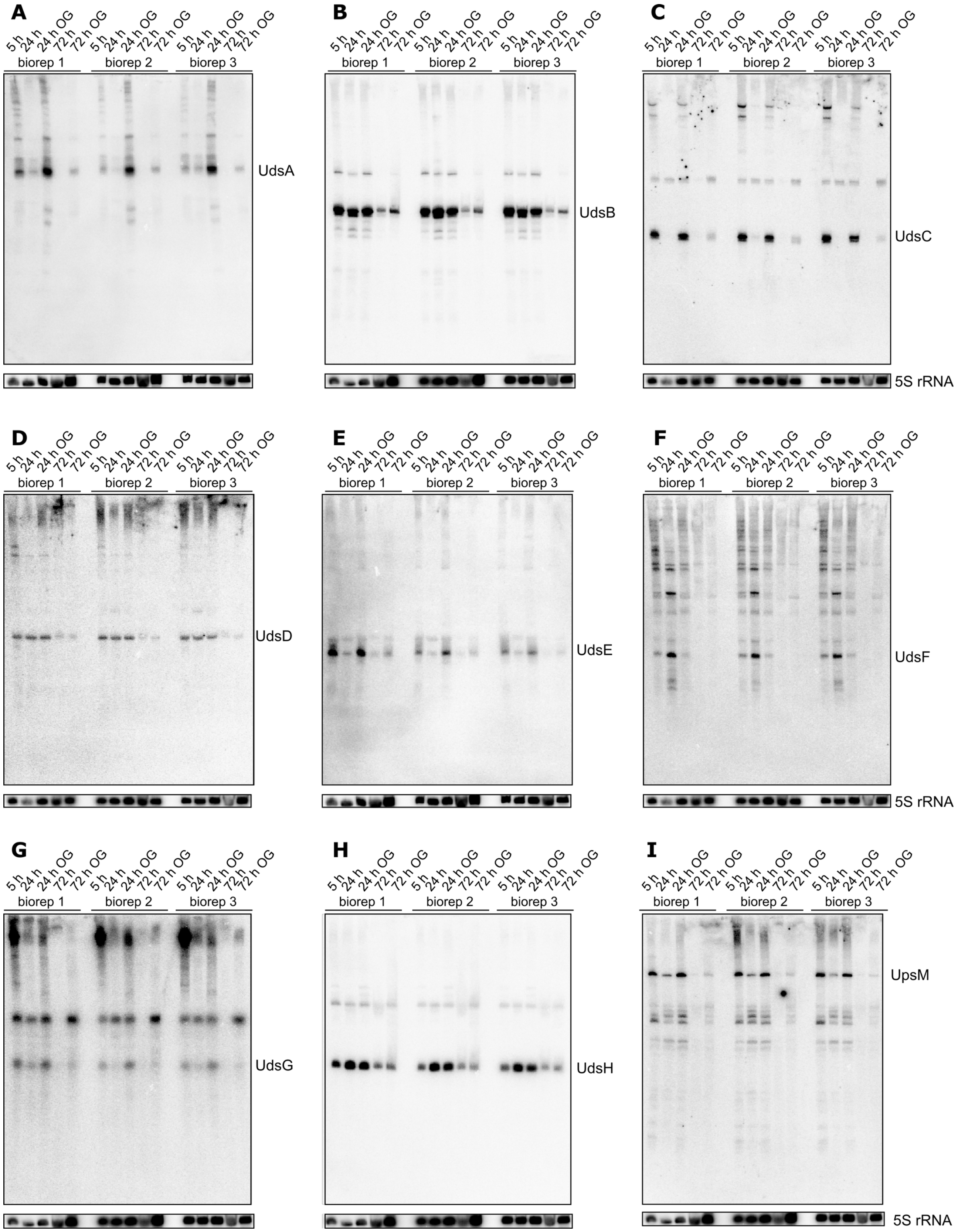

Figure S5. Northern blots of the analyzed UTR-derived sRNAs during different growth phases. Liquid cultures of the *R. sphaeroides* wildtype were incubated for 72 h under microaerobic conditions (biological triplicates). Cell samples were taken after 5 h, 24 h and 72 h. Moreover, outgrowth cultures were inoculated after 24 h and 72 h and cultivated for 1 h before harvesting. Total RNA was isolated and a northern blot analysis performed with specific probes directed against the UdsA (**A**), UdsB (**B**), UdsC (**C**), UdsD (**D**), UdsE (**E**), UdsF (**F**), UdsG (**G**), UdsH (**H**) and UpsM (**I**). Loading control: 5S rRNA.

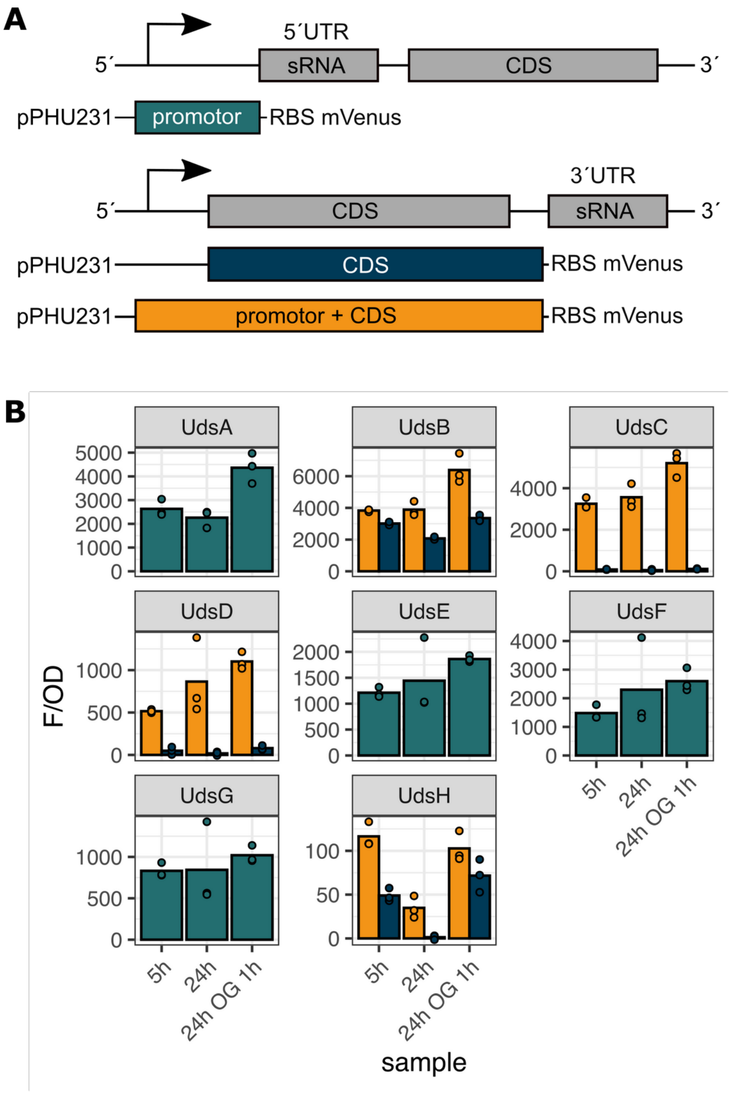

Figure S6. The UTR-derived sRNA promoter activity is growth phase dependent. The plasmids with indicated sequences were conjugated in the wild type strain and the fluorescence intensity was measured after 5 h, 24 h and from the outgrowth culture. x-axis: sample. y-axis: F/OD$_{660\ nm}$. Green: promoter sequence. Blue: coding sequence (CDS). Yellow: promoter + coding sequence. n = 3.

Every dot represents the mean value of two technical replicates.

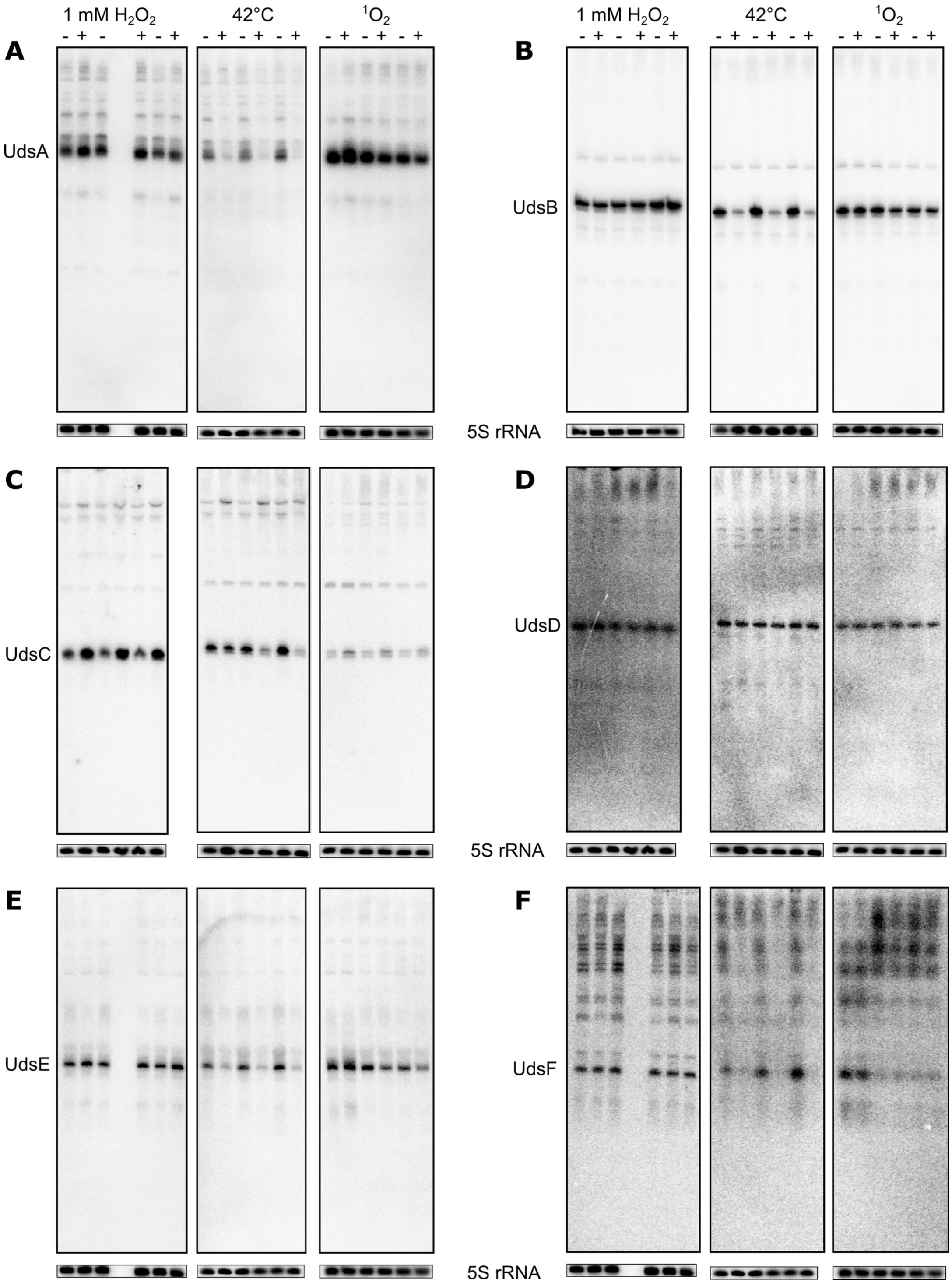

Figure S7. UTR-derived sRNA levels are modulated by different stress conditions. Exponentially growing *R. sphaeroides* liquid cultures were exposed to oxidative (1 mM H_2O_2, 10 min), heat (42 °C, 30 min) or singlet oxygen stress ($^1O_2$, 10 min). Samples for RNA isolation were harvested before and after the indicated timepoints. Northern blot analysis, biological triplicates, 10 µg total RNA per lane. Total RNA was isolated and a northern blot analysis performed with specific probes directed against the UdsA (**A**), UdsB (**B**), UdsC (**C**), UdsD (**D**), UdsE (**E**) and UdsF (**F**). Loading control: 5S rRNA.

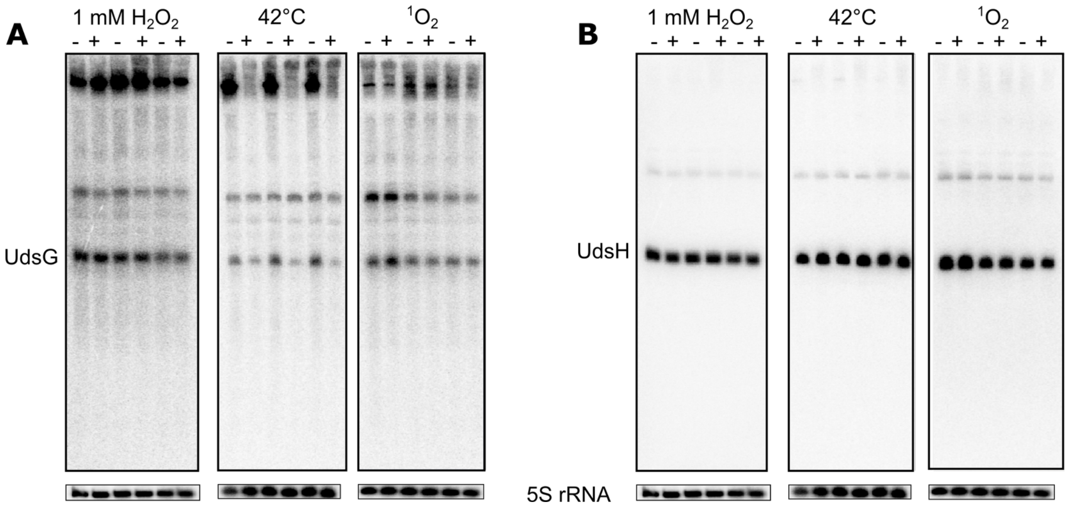

Figure S8. UTR-derived sRNA levels are modulated by different stress conditions. Exponentially growing *R. sphaeroides* liquid cultures were exposed to oxidative (1 mM H_2O_2, 10 min), heat (42 °C, 30 min) or singlet oxygen stress ($^1O_2$, 10 min). Samples for RNA isolation were harvested before and after the indicated timepoints. Northern blot analysis, biological triplicates, 10 µg total RNA per lane. Total RNA was isolated and a northern blot analysis performed with specific probes directed against the UdsG (**A**), UdsH (**B**). Loading control: 5S rRNA.

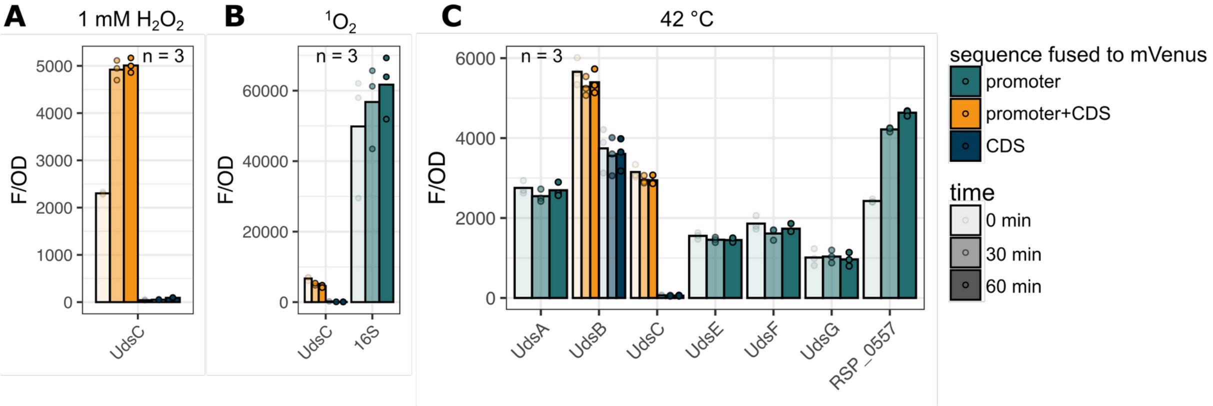

Figure S9. Normalized fluorescence intensity of the wild type harbouring the indicated plasmids before and after induction with 1 mM H_2O_2 (**A**), $^1O_2$ stress (**B**), 42 °C heat stress(**C**). x-axis: sample. y-axis: $F/OD_{660\ nm}$. Green: promoter sequence. Blue: coding sequence (CDS). Yellow: promoter + coding sequence. Color intensity indicates duration of induction. n = 3. Every dot represents the mean value of two technical replicates. The promoters of *RSP_0557* (unpublished data) and 16S rRNA (McIntosh et al., 2019) were used as positive controls for the indicated growth conditions.

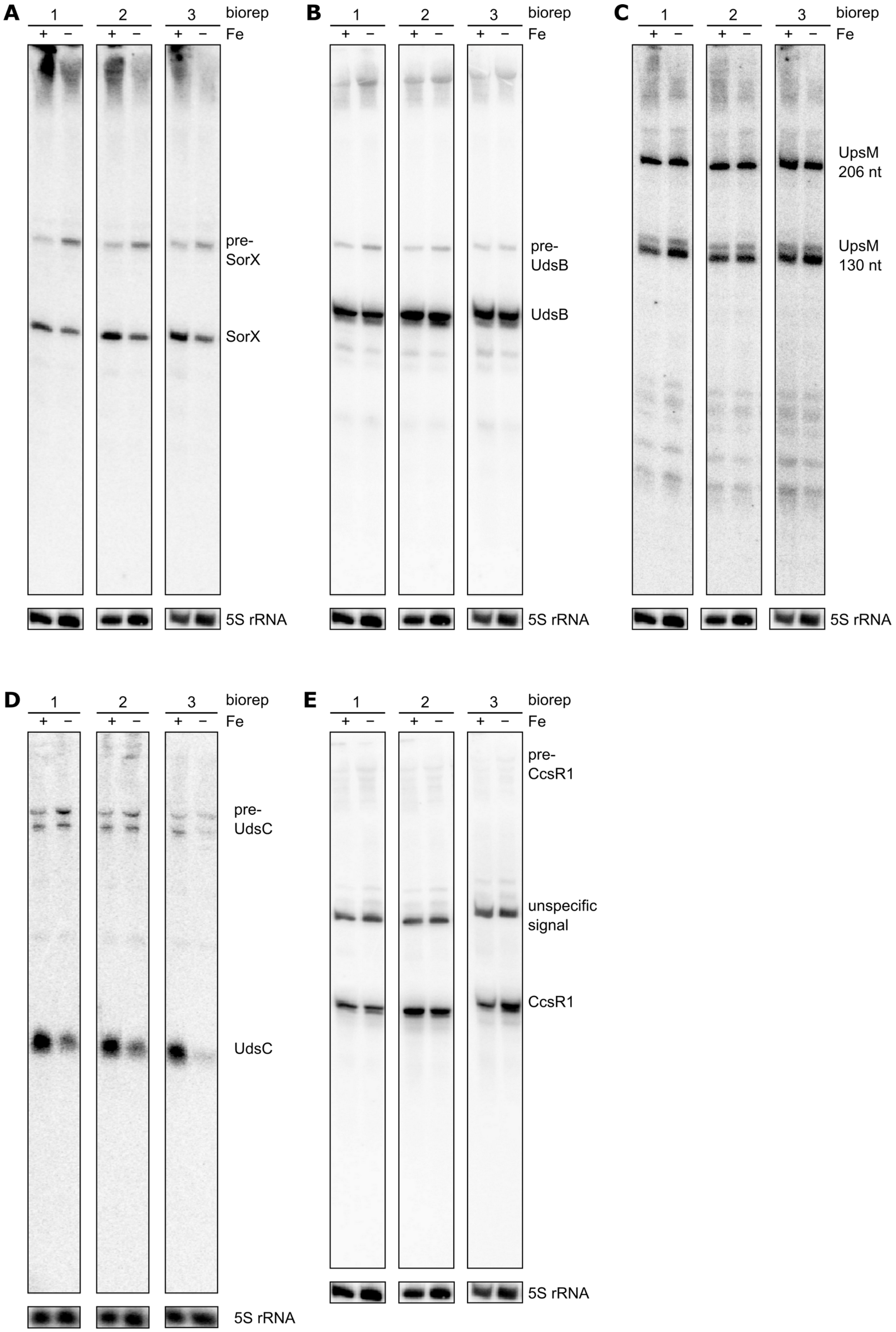

Figure S10. Total RNA from *R. sphaeroides* wild type cultures grown in media supplemented with iron (+Fe) or after iron depletion (-Fe) was isolated and analyzed via northern blot. Membranes were hybridized with probes directed against sRNAs which are processed by RNase E: SorX (**A**), UdsB (**B**), UpsM (**C**), UdsC (**D**) and CcsR1 (**E**). The experiment was performed in biological triplicates. 5S rRNA served as loading control.

Table S1. Promotor sequences of the UTR derived sRNAs characterized in this study. Bold typset indicates the -35 and -10 regions.

sRNA	promoter sequence	
UdsA	**TTG**ACATCCCCTCCGACTCCCC**CTATAC**GCCGCC	RpoH$_I$/H$_{II}$
UdsB	**TTG**CGCTTCGGTTCGCAATATTATACCGAATTCGGCC	-
UdsC	**TGT**GCTTGGCGCAGCAAGGGG**CTAGGT**GTTGCCGC	RpoH$_{II}$
UdsD	**TCA**AGGAACAAAGCCCCATTCGATCACACATTCGGGGT	-
UdsE	**TTG**CGTCCCGGCCGGAATGGGC**CTATAC**GCCCTTCC	possible RpoH$_I$/H$_{II}$, imperfect consensus sequence
UdsF	**TTG**ATTGTATCTTAATGAAACT**CTATCG**CTAAAGT	possible RpoH$_I$/H$_{II}$, imperfect consensus sequence
UdsG	**TTG**CTCGACACGACGGAACTGA**CTATAG**ACGCCGC	-
UdsH	**TTG**CCTGACACGCCGCTTCGAC**CCATGT**TCCGGAGC	RpoH$_I$/H$_{II}$

Table S2. Strains used in this study.

Rhodobacter sphaeroides strain	genotype	reference
2.4.1	wild type	van Niel, 1944
rne^{ts}	Δrne::rne$^{E.c.ts}$;Sp	Weber et al., 2016
Δ*rnc*	Δ(rnc::Km)	Rische-Grahl et al., 2014
*pnp*ΔKH-S1	Δpnp_KH-S1::Gm	Spanka et al., 2021
Δ*rnj*	Δrnj::Km	Rische and Klug, 2012
Δ*rppH*	deletion of the putative RNA pyrophosphohydrolase RppH (RSP 0931, *ialA*)	this study
Δ*ybeY*	ΔRSP_3598:Gm	this study
Δ*hfq*	hfq::ΩSp	Glaeser et al., 2007

Table S3. List of all plasmides used in this study.

pPHU231_P16S_mVenus	promoter of 16S rRNA	McIntosh et al., 2019
pPHU231_PudsA_mVenus	promoter of *udsA* (CDS of RSP_1711)	this study
pPHU231_PudsB_full_mVenus	promoter + CDS of *RSP_1771* (upstream of *udsB*)	this study
pPHU231_PudsB_CDS_mVenus	CDS of *RSP_1771* (upstream of *udsB*)	this study
pPHU231_PudsC_full_mVenus	promoter + CDS of *RSP_7527* (upstream of *udsC*)	this study
pPHU231_PudsC_CDS_mVenus	CDS of *RSP_7527* (upstream of *udsC*)	this study
pPHU231_PudsD_full_mVenus	promoter + CDS of *RSP_0697* (upstream of *udsD*)	this study
pPHU231_PudsD_CDS_mVenus	CDS of *RSP_0697* (upstream of *udsD*)	this study
pPHU231_PudsE_mVenus	promoter of *udsE* (upstream of RSP_1162)	this study
pPHU231_PudsF_mVenus	promoter of *udsF* (upstream of RSP_6236)	this study
pPHU231_PudsG_mVenus	promoter of *udsG* (upstream of RSP_3590)	this study
pPHU231_PudsH_full_mVenus	promoter + CDS of *RSP_3594* (upstream of *udsH*)	this study
pPHU231_PudsH_CDS_mVenus	CDS of *RSP_3594* (upstream of *udsH*)	this study

Table S4. List of all oligonucleotides used in this study.

oligonucleotide	5´→ 3´ sequence	reference	purpose
probe_RSP1771_3UTR	GCCTCCCAACTTACCGCAGCTCCGG	this study	northern blot
probe_RSP0697_3UTR	GCGAGGCGGGCAGAGGCAGCCCCTT	this study	northern blot
probe_RSP1162_5UTR	TGCGATCATCGGTGGAGCCATCGGT	this study	northern blot
probe_rpsL_5UTR	CGGATCATGACCACTACAGTTAACT	this study	northern blot
probe_RSP7527_3UTR	CTGCGACAATGACTGCCTCAGCCGA	this study	northern blot
probe_RSP3594_3UTR	ACTCTTGCTGAAAAGGCGCTTCGTG	this study	northern blot
probe_RSP6236_5UTR	ACCAGTTCGTCTTCCGGCAGTATCA	this study	northern blot
probe_RSP3590_5UTR	CGACCCTGCGCCTGCCTCAGTTCGA	this study	northern blot
p-5S	CTTGAGACGCAGTACCATTG	Berghoff et al., 2009	northern blot
p-tRNA-Ala	GAGCCTATCGGGATCGAAC	Berghoff et al., 2009	northern blot
p-tmRNA	GAACTGCTTACGCAGCCAG	Berghoff et al., 2009	northern blot
p_0847_SorX_for	ACGAAGGAGTGACCGATGG	Peng et al., 2016	northern blot
p_0847_SorX_rev	AAAAAGGCCGGGCACGAG	Peng et al., 2016	northern blot
CcsR1	CGTCGCCGCTGCTGCTACAGGTC	Billenkamp et al., 2015	northern blot
UpsM	GACTCAGGTGGTCGCCAGATACC	Weber et al., 2016	cloning
KO_RSP0931_up_f	GCAACCGGTCGGAATTCGACCTGC	this study	cloning
KO_RSP0931_up_r	GCCCGGCGAGGATCCGACCCTCG	this study	cloning
KO_RSP0931_dw_f	GGTCTATGAGGATCCGGTGGCGACC	this study	cloning
KO_RSP0931_dw_r	GCGCCTCCAAGCTTTCTCGATCC	this study	cloning
KO_3598_ybeY_up_f	ATGAATTCCCCTCTCGAACGCCTTCG	this study	cloning
KO_3598_ybeY_up_r	TAGGATCCTCCGCCGGCATGTCCAGC	this study	cloning
KO_3598_ybeY_dw_f	ATGGATCCAGGCGGACGGCGACCTGATGG	this study	cloning
KO_3598_ybeY_dw_r	TAGCATGCGGCACGGCCACGATCTCG	this study	cloning
1771_Pro_Hind_for	actaAAGCTTgccgaggaagagcaggta	this study	promotor fusion
1771_Pro_RBS_Xba_rev	actatctagacttctCCCCTgcggtctctcggcaagg	this study	promotor fusion
3590_Pro_Hind_for	actaAAGCTTttcacggtggcgatcgac	this study	promotor fusion
3590_Pro_RBS_Xba_rev	actaTCTAGActtctCCCCTtgcggcgtctatagtcagtt	this study	promotor fusion
6236_Pro_Hind_for	actaAAGCTTggagtcaggatcagctcg	this study	promotor fusion
6236_Pro_RBS_Xba_rev	actaTCTAGActtctCCCCTtactttagcgatagagtttcattaaga	this study	promotor fusion
0697_CDS_Hind_for	actaAAGCTTgcctataaatccttgctgacggt	this study	promotor fusion
0697_CDS_RBS_Xba_rev	actaTCTAGActtctCCCCTttccgtcagtgcgccatc	this study	promotor fusion
0697_Pro_Hind_for	actaAAGCTTgcacctgccacaggaaca	this study	promotor fusion
0697_Pro_RBS_Xba_rev	actaTCTAGActtctCCCCTctcacaacctccagccgat	this study	promotor fusion
3594_full_Hind_for	cgaAAGCTTcgtcactcatttcggcacct	this study	promotor fusion
3594_Pro_RBS_Xba_rev	actaTCTAGActtctCCCCTctctgtctccgttgttcgcg	this study	promotor fusion
3594_coding_Hind_for	cgaAAGCTTatgaccatcgaccttcgcaaga	this study	promotor fusion
3594_sRNA_Xba_RBS_rev	cagttctagacttctCCCCTgcgcttcgtgaaatccggttc	this study	promotor fusion
1771_CDS_Hind_for	actaAAGCTTatgagcttccgccttcagc	this study	promotor fusion
1771_CDS_RBS_Xba_rev	actaTCTAGActtctCCCCTtcaggccgagatcatttctgc	this study	promotor fusion
1162_UTR_ScaI_for	cagAGTACTtcgccttcacctcgaaatcgg	this study	promotor fusion
1162_Pr_Xba_RBS_rev	cagttctagacttctCCCCTggaagggcgtataggccca	this study	promotor fusion
7527_full_Hind_for	cgaAAGCTTggcctcgatggctggaga	this study	promotor fusion
7527_coding_Hind_for	cgaAAGCTTatgacccggacgaatggaca	this study	promotor fusion
7527_sRNA_Xba_RBS_rev	cagttctagacttctCCCCTtcctcgtgcgctcatgcc	this study	promotor fusion

1711_coding_Hind_for	cgaAAGCTTgtgcgactctcggacaatctg	this study	promotor fusion
1711_sRNA_XbaI_RBS_rev	cagttctagacttctCCCCTtaggcggcgtatagggga	this study	promotor fusion
1771_mRNA_for	cgccacggtctacaagggaa	this study	RT-PCR
1771_sRNA_rev	ttgcctcccaacttaccgca	this study	RT-PCR
0697_mRNA_for	tctggtgatgggtgcctacg	this study	RT-PCR
0697_sRNA_rev	cagaggcagccccttcttc	this study	RT-PCR
1162_sRNA_for_2	gcctcggcttccaaaaccg	this study	RT-PCR
1162_mRNA_rev	gtcttgccgctcatcagcc	this study	RT-PCR
6236_mRNA_rev	acgttgcgaaaaccggtcat	this study	RT-PCR
6236_sRNA_for	gttacaacctttagttgatactgccgga	this study	RT-PCR
3590_sRNA_for	ggcgatcgaagccatcgaac	this study	RT-PCR
3590_mRNA_rev	catggttgctttagcgcacat	this study	RT-PCR
3591_mRNA_for	ctggccgatctgcgcgag	this study	RT-PCR
3590_sRNA_rev	cacggcaatgagggccga	this study	RT-PCR
1711_sRNA_rev	cactacagttaactgaagctggctgc	this study	RT-PCR
1711_coding_for	gtgcgactctcggacaatctg	this study	RT-PCR
1711_sRNA_for	agttaactgtagtggtcatgatccg	this study	RT-PCR
rpsL_coding_start_rev	atgagctgttggatcgttggcat	this study	RT-PCR
7527_coding_for	atgacccggacgaatggaca	this study	RT-PCR
7527_sRNA_rev	tccatgctgcgacaatgactgc	this study	RT-PCR
3594_gap_for	tgcctatgcccgcgaca	this study	RT-PCR
3594_gap_rev	gtcgttctgaaactcttgctgaaaagg	this study	RT-PCR

References

Glaeser J, Zobawa M, Lottspeich F, Klug G. Protein synthesis patterns reveal a complex regulatory response to singlet oxygen in Rhodobacter. J Proteome Res. 2007 Jul;6(7):2460-71. doi: 10.1021/pr060624p. Epub 2007 May 31. PMID: 17536848.

McIntosh M, Eisenhardt K, Remes B, Konzer A, Klug G. Adaptation of the Alphaproteobacterium Rhodobacter sphaeroides to stationary phase. Environ Microbiol. 2019 Nov;21(11):4425-4445. doi: 10.1111/1462-2920.14809. Epub 2019 Oct 17. PMID: 31579997.

Peng T, Berghoff BA, Oh JI, Weber L, Schirmer J, Schwarz J, Glaeser J, Klug G. Regulation of a polyamine transporter by the conserved 3' UTR-derived sRNA SorX confers resistance to singlet oxygen and organic hydroperoxides in Rhodobacter sphaeroides. RNA Biol. 2016 Oct 2;13(10):988-999. doi: 10.1080/15476286.2016.1212152. Epub 2016 Jul 15. PMID: 27420112; PMCID: PMC5056773.

Rische T, Klug G. The ordered processing of intervening sequences in 23S rRNA of Rhodobacter sphaeroides requires RNase J. RNA Biol. 2012 Mar;9(3):343-50. doi: 10.4161/rna.19433. Epub 2012 Mar 1. PMID: 22336705.

Rische-Grahl T, Weber L, Remes B, Förstner KU, Klug G. RNase J is required for processing of a small number of RNAs in Rhodobacter sphaeroides. RNA Biol. 2014;11(7):855-64. doi: 10.4161/rna.29440. Epub 2014 Jun 12. PMID: 24922065; PMCID: PMC4179960.

Spanka DT, Reuscher CM, Klug G. Impact of PNPase on the transcriptome of Rhodobacter sphaeroides and its cooperation with RNase III and RNase E. BMC Genomics. 2021 Feb 6;22(1):106. doi: 10.1186/s12864-021-07409-4. PMID: 33549057; PMCID: PMC7866481.

Weber L, Thoelken C, Volk M, Remes B, Lechner M, Klug G. The Conserved Dcw Gene Cluster of R. sphaeroides Is Preceded by an Uncommonly Extended 5' Leader Featuring the sRNA UpsM. PLoS One. 2016 Nov 1;11(11):e0165694. doi: 10.1371/journal.pone.0165694. PMID: 27802301; PMCID: PMC5089854.

van Niel C. B. The culture, general physiology, morphology, and classification of the non-sulfur purple and brown bacteria. Microbiology and Molecular Biology Reviews, 8(1):1–118, 1944. ISSN 0005-3678. URL https://mmbr.asm.org/content/8/1/1.
